# *Anopheles gambiae* PGRPLC-Mediated Defense against Bacteria Modulates Infections with Malaria Parasites

**DOI:** 10.1371/journal.ppat.1000542

**Published:** 2009-08-07

**Authors:** Stephan Meister, Bogos Agianian, Fanny Turlure, Angela Relógio, Isabelle Morlais, Fotis C. Kafatos, George K. Christophides

**Affiliations:** 1 Division of Cell and Molecular Biology, Department of Life Sciences, Imperial College London, London, United Kingdom; 2 Department of Molecular Biology and Genetics, Democritus University of Thrace, Alexandropolis, Greece; 3 European Molecular Biology Laboratory, Heidelberg, Germany; 4 Organisation de Coordination de la lutte contre les Endémies en Afrique Centrale, Laboratoire de Recherche sur le Paludisme, Yaoundé, Cameroon; 5 Institut de Recherche pour le Développement - Laboratoire de Lutte contre les Insectes Nuisibles, UR 016, BP 64501, Montpellier, France; Stanford University, United States of America

## Abstract

Recognition of peptidoglycan (PGN) is paramount for insect antibacterial defenses. In the fruit fly *Drosophila melanogaster*, the transmembrane PGN Recognition Protein LC (PGRP-LC) is a receptor of the Imd signaling pathway that is activated after infection with bacteria, mainly Gram-negative (Gram−). Here we demonstrate that bacterial infections of the malaria mosquito *Anopheles gambiae* are sensed by the orthologous PGRPLC protein which then activates a signaling pathway that involves the Rel/NF-κB transcription factor REL2. PGRPLC signaling leads to transcriptional induction of antimicrobial peptides at early stages of hemolymph infections with the Gram-positive (Gram+) bacterium S*taphylococcus aureus*, but a different signaling pathway might be used in infections with the Gram− bacterium *Escherichia coli*. The size of mosquito symbiotic bacteria populations and their dramatic proliferation after a bloodmeal, as well as intestinal bacterial infections, are also controlled by PGRPLC signaling. We show that this defense response modulates mosquito infection intensities with malaria parasites, both the rodent model parasite, *Plasmodium berghei*, and field isolates of the human parasite, *Plasmodium falciparum*. We propose that the tripartite interaction between mosquito microbial communities, PGRPLC-mediated antibacterial defense and infections with *Plasmodium* can be exploited in future interventions aiming to control malaria transmission. Molecular analysis and structural modeling provided mechanistic insights for the function of PGRPLC. Alternative splicing of *PGRPLC* transcripts produces three main isoforms, of which PGRPLC3 appears to have a key role in the resistance to bacteria and modulation of *Plasmodium* infections. Structural modeling indicates that PGRPLC3 is capable of binding monomeric PGN muropeptides but unable to initiate dimerization with other isoforms. A dual role of this isoform is hypothesized: it sequesters monomeric PGN dampening weak signals and locks other PGRPLC isoforms in binary immunostimulatory complexes further enhancing strong signals.

## Author Summary

Recognition of peptidoglycan on the bacteria cell wall triggers insect immune responses. The fruit fly PGRPLC receptor protein senses the presence of peptidoglycan and activates a pathway that mediates resistance to bacterial infections, mainly Gram-negative. We show that the PGRPLC receptor of the malaria vector mosquito *Anopheles gambiae* can also sense infections of the hemolymph (the mosquito blood) or the gut with bacteria of both Gram types and thereby activate a pathway that confers resistance to these infections. PGRPLC and its downstream responses also control the numbers of symbiotic bacteria that are mostly found in the mosquito gut where they drastically proliferate after a female mosquito takes a bloodmeal. Importantly, when the bloodmeal is infected with malaria parasites, the defense reaction that the mosquito mounts against proliferating bacteria also eliminate a large number of parasites. These mechanisms are largely elucidated using a rodent malaria parasite, but we also show that they significantly affect the intensities of mosquito infections with *Plasmodium falciparum* parasites found in the blood of children in sub-Saharan Africa.

## Introduction

Immune signaling is triggered by recognition of molecular patterns that are common in microbes but absent from the host. PGN is a cell wall component of Gram+ and Gram− bacteria and bacilli, but its amount, sub-cellular localization and specific composition vary between different bacteria, and may set the basis for specific recognition by PGN recognition proteins such as PGRPs. These proteins share a conserved PGRP domain that is similar to the T7 lysozyme.

The *Drosophila melanogaster* PGRP-SA [1] and PGRP-SD [2] are essential for activation of Toll signaling. In contrast, PGRP-LC [3],[4] and PGRP-LE [5] trigger Imd pathway activation. The *PGRP-LC* gene encodes three PGRP ectodomains, each of which fuses by alternative splicing to an invariant part, generating three distinct isoforms: PGRP-LCx, -LCy and -LCa. The intracellular invariant part encompasses an IMD interaction domain and a receptor-interacting protein homotypic interaction motif (RHIM)-like motif, which mediate contact with the IMD receptor-adaptor protein [6] and perhaps an unknown factor, respectively [5], to initiate signal transduction.

Several studies have provided novel, important insights into the structural basis of PGN recognition by PGRPs. Crystal structures have been determined for six *Drosophila* PGRPs [7],[8],[9],[10],[11],[12],[13], including PGRP-LE and the heterodimer PGRP-LCx/LCa in complex with monomeric *meso*-diaminopimelic acid (DAP)-type PGN, which is released mostly from Gram− bacteria during PGN turnover and is known as tracheal cytotoxin (TCT). These structures suggest that PGRP-LCx is sufficient for Imd pathway activation by polymeric DAP-type PGN, whereas heterodimerization with PGRP-LCa is required for response to monomeric PGN [14],[15]. PGRP-LCa itself is unable to bind PGN and its suggested role is to “lock” PGRP-LCx in a monomeric PGN binding mode.

*Anopheles gambiae*, the major mosquito vector of human malaria in Africa, encodes seven PGRPs, five of which (LA, LB, LC, LD and S1) are orthologous to *Drosophila* PGRPs [16]. Similar to its fly ortholog, PGRPLC encompasses three PGRP domains (LC1, LC2 and LC3) that are utilized via alternative splicing for production of three main protein isoforms [16],[17]. Here, we investigate the role of PGRPLC in mosquito infections with bacteria and malaria parasites. Theoretical structural modeling indicates that PGRPLC can recognize PGN from both Gram+ *Staphylococcus aureus* and Gram− *Escherichia coli* bacteria, and experimental results demonstrate that indeed PGRPLC mediates resistance against such infections. PGRPLC3 is a key modulator of these reactions. The structural modeling data suggest that, upon monomeric PGN binding, PGRPLC3 may lock other PGRPLC isoforms in binary immunostimulatory complexes, through a mechanism that differs significantly from that employed by *Drosophila* PGRP-LCa. PGRPLC3 can also sequester monomeric PGN perhaps to prevent unnecessary immune activation during low infections. Importantly, PGRPLC signaling modulates the intensity of mosquito infections with human and rodent malaria parasites. We also demonstrate that PGRPLC initiates responses against microbiota and bacterial infections of the midgut. In female mosquitoes, the size of the midgut bacterial communities substantially increase after a bloodmeal, causing further activation of PGRPLC signaling that appears to consequently affect the parasite infection intensities.

## Results

### *PGRPLC* is required for resistance to bacterial infections

**Figure 1:**
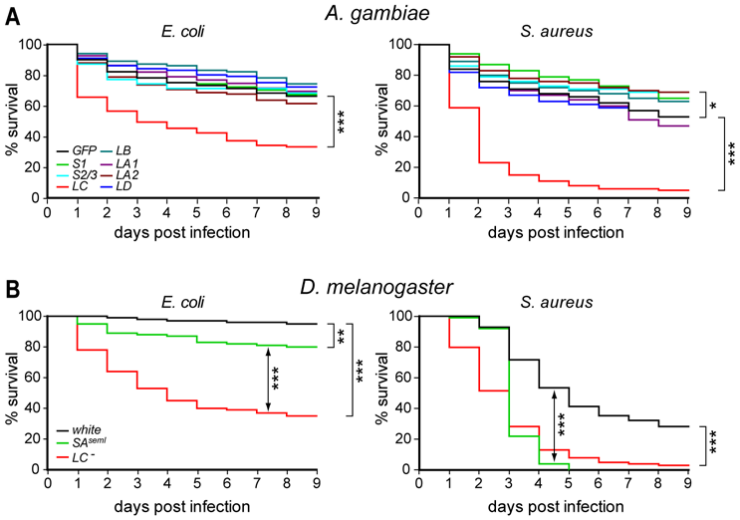
Resistance of PGRP kd mosquitoes and mutant fruit flies to bacterial infections. (A) Kaplan Meier survival curves of adult *A. gambiae* females silenced for *PGRP* gene expression, and infected 4 days later with *E. coli* (left) or *S. aureus* (right). *PGRPS2* and *S3* were concomitantly silenced using dsRNA that fully matched both sequences. Survival was recorded daily for 9 days and compared to that of *GFP* dsRNA-injected controls. The data are the average of very similar results obtained from at least three replicate infections. (B) Kaplan Meier survival curves of adult *D. melanogaster* females of the *PGRP-SA^seml^*, *PGRP-LC^−^* and *white* (control) mutant strains infected with *E. coli* (left) or *S. aureus* (right).

We injected dsRNA into newly emerged adult female *A. gambiae* to silence by RNAi the expression of corresponding *PGRP* genes. Four days later, the mosquitoes were infected with *E. coli* or *S. aureus*, two bacteria species with different types of PGN: DAP and Lysine (Lys)-type PGN, respectively. The survival of these mosquitoes was monitored daily and compared to the survival of *GFP* dsRNA-injected controls using Log-rank and Gehan-Breslow-Wilcoxon tests of survival curves. *PGRPLC* silencing had a pronounced effect (P<0.001 with both tests) on survival after infections with either bacterium (Figure 1A). *E. coli* infections killed 50% of the *PGRPLC* knockdown (kd) mosquitoes by day 4 and subsided thereafter. *S. aureus* infections killed 50% of the kd mosquitoes by day 2 and almost all mosquitoes by day 6. Interestingly, *PGRPLA2* silencing had a weak but significant (P<0.05) protective effect against *S. aureus* infections. *PGRPS1* and *PGRPS2/3* kds also exerted minor protective effects that were statistically significant (P<0.05) only with one of the two tests (*PGRPS2* and *S3* were silenced simultaneously as their sequences are almost identical). Injection with *dsGFP* or saline alone (Figure S1) did not cause mosquito mortality, indicating that the documented phenotypes were indeed due to the combination of specific *PGRP* gene silencing and exogenous bacterial infections.

These data indicated a potential difference in the PGRPLC-mediated antibacterial defense between *Anopheles* and *Drosophila*, which we further investigated by subjecting *D. melanogaster PGRP-LC^−^* mutants [3] to our infection assays. *PGRP-SA^seml^* [1] and *white* mutant strains were used as controls. Two to three day-old flies were injected with *E. coli* or *S. aureus* and their mortality rates were recorded daily and compared (Figure 1B). *PGRP-LC^−^* flies displayed pronounced mortality (P<0.001) following *E. coli* infection, which reached 50% by day 3. Mortality of *PGRP-SA^seml^* flies following *E. coli* infection was markedly less but also significant (P<0.01). Similarly, both *PGRP-LC^−^* and *PGRP-SA^seml^* flies showed a significant drop (P<0.001) in survival when infected with *S. aureus*, which reached 50% at day 3 and over 90% at day 5. Importantly, the survival curves suggested that the presence of PGRP-LC sufficed to contain *S. aureus* infection in *PGRP-SA^seml^* mutants in the first 3 days after the infection, but thereafter PGRP-SA was indispensable. *PGRP* mutant and control *white* flies survived equally well when injected with saline alone (data not shown).

### Modular PGRPLC receptors are generated through alternative splicing

**Figure 2:**
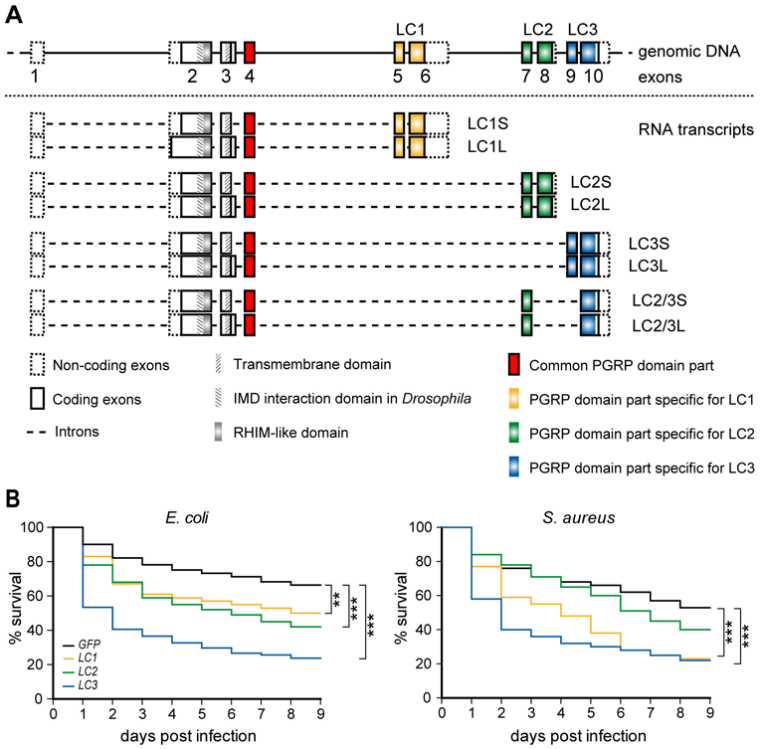
Contribution of *A. gambiae* PGRPLC isoforms generated through alterative splicing in antibacterial defense. (A) Genomic organization of the *PGRPLC* locus and alternative splice variants identified through genomic PCR and RT-PCR reactions. Sequences encoding variable parts of the 3 PGRP domains and the sequence encoding the common part of all PGRP domains are depicted in different colors. (B) Percent survival rates of *A. gambiae* females silenced for the expression of each of the 3 main PGRPLC isoforms or all isoforms simultaneously and infected 4 days later with *E. coli* (left) or *S. aureus* (right). Survival was recorded daily, for 5 days. *GFP* dsRNA-injected controls were used as controls. Colors corresponding to those used to indicate different isoforms in (A). Error bars represent standard errors of three or more replicate infections.

We investigated the complex architecture of the *PGRPLC* gene, by mapping on the *A. gambiae* genome the sequences of all available related ESTs and various genomic PCR or RT-PCR reactions. The relative positions of the primers used in these reactions are shown in Figure S2 and their sequences are listed in Table S1. As shown previously [16],[17], *PGRPLC* encodes three main protein isoforms (LC1, LC2 and LC3), each having a different PGRP domain and an optional 75-nucleotide cassette at the 3′ end of the common exon 3 (Figure 2A). Thus, each isoform exists in two versions; the long one (-L) is 25 amino acids (aa) longer than the short version (-S), which utilizes a cryptic splice acceptor in exon 3. Sequence alignment of the *Anopheles* (*Ag*) and *Drosophila* (*Dm*) PGRPLCs (Figure S3) and mapping of available related ESTs on the *Drosophila* genome revealed an equivalent albeit shorter (57 nucleotide) optional cassette in the *DmPGRP-LC* gene. In both insects, these cassettes are extracellular and located immediately downstream of the putative transmembrane domain. Use of the DisEMBL Intrinsic Protein Disorder Prediction algorithm (http://dis.embl.de/) predicted that they encode a hot loop region, a flexible structure that could be important in protein interactions.

Each PGRP domain of *Ag*PGRPLC is encoded by three exons: the common exon 4 and two variable exons. The introns separating these variable exons are at identical relative positions suggesting that additional hybrid domains could be generated through alternative splicing. We tested this hypothesis by RT-PCR using combinations of primers distributed along the *PGRPLC* gene (Figure S2 and Table S1). Indeed, we detected a novel transcript encoding a hybrid PGRPLC2/3 domain, also showing optional association with the 25-aa cassette (Figure 2A). Additional exon combinations were detected (Figure S4), but they exhibited frameshifts leading to premature stop codons or presumably non-functional domains. In addition, we detected non-random transcripts encompassing unspliced versions of *PGRPLC1* and *LC2* (Figure S4). In contrast, the most abundantly expressed *PGRPLC3* transcript showed no unspliced versions in either the EST databases or the cDNA products.

### PGRPLC3 is essential for antibacterial immunity

We examined the contribution of each of the three main *Ag*PGRPLC isoforms to antibacterial defense by silencing each one independently in adult *Anopheles* mosquitoes that were infected 4 days later with *E. coli* or *S. aureus*. Quantitative real time-PCR (qRT-PCR) showed isoform-specific silencing that varied quantitatively between isoforms: 65% for *LC1*, and 30% for *LC2* and *LC3* (Figure S5). These levels were comparable with those obtained after silencing the entire *PGRPLC* gene by targeting common exons 2–4 as above. The survival of mosquitoes after bacterial infections was recorded daily for 9 days and referenced to the survival of infected control mosquitoes injected with *GFP* dsRNA (Figure 2B). *E. coli* infections significantly reduced the survival of *LC3* (P<0.001; 50% at day 2) and to a lesser extent *LC1* or *LC2* (40–50% at day 6; P<0.05 and P<0.001, respectively) kd mosquitoes. Susceptibility of mosquitoes to *S. aureus* infections was significantly reduced (P<0.001) after silencing *LC3* (50% at day 1) and *LC1* (50% at day 5); silencing *LC2* appeared to have a minor effect on mosquito survival, which was not statistically significant. These data in conjunction with the silencing efficiency indicated that PGRPLC3 might be the most important isoform in the antibacterial defense.

### PGRPLC regulates AMP expression at early stages of *S. aureus* infections

**Figure 3:**
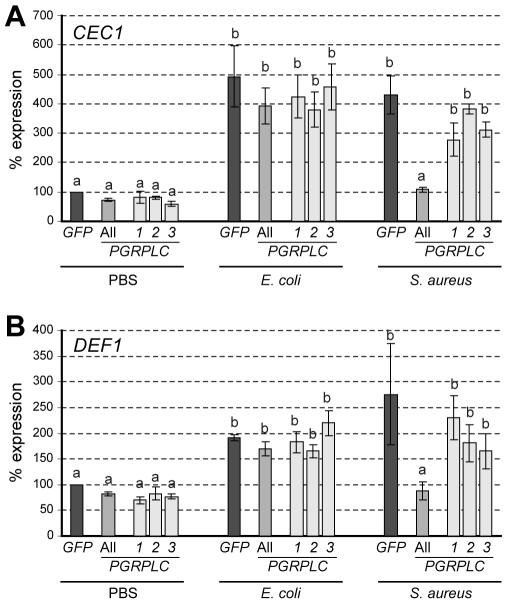
Role of PGRPLC in AMP expression following bacterial infections. Relative percent abundance of *CEC1* (A) and *DEF1* (B) transcripts in *PGRPLC* kd and ds*GFP*-treated control *A. gambiae* adult females, 3 h after injection with saline solution (PBS), *E. coli* or *S. aureus*. Error bars indicate standard deviations. Same letters above each bar represent statistically similar expression values while different letters indicate statistically significant differences (P<0.001) as determined by multiple comparisons using the Student's t-test.

Initial experiments indicated that mosquito antimicrobial peptide (AMP) genes are transcriptionally induced as early as 3 h after *E. coli* or *S. aureus* infections but not after saline injections alone (data not shown). We examined whether PGRPLC is involved in this response by comparing the levels of *CEC1* and *DEF1* transcripts in *PGRPLC* kd and ds*GFP*-treated control mosquitoes, 3 h after injection with bacteria or saline (Figure 3). Uninfected mosquitoes exhibited basal expression levels of both *AMPs*, which were slightly reduced after silencing *PGRPLC*. Infections with either bacterium induced the expression of both *CEC1* and *DEF1*, 4–5 and 2–3 fold, respectively. However, this induction was PGRPLC-mediated only in *S. aureus* infections, not in *E. coli*. Silencing separately the three main isoforms did not reproduce the effect of silencing the entire gene although a previous study showed that overexpression of PGRPLC1 or LC3 in cultured cells leads to induction of *CEC1* expression [17]. Maybe this was partly due to the low level silencing of the individual isoforms.

Previous studies have shown that *Drosophila* and *Anopheles* PGRPLCs act as phagocytic receptors of *E. coli* [18],[19]. Since the transcriptional induction of *CEC1* or *DEF1* at early phases of *E. coli* infections was PGRPLC-independent, we examined whether PGRPLC confers resistance to *E. coli* by phagocytosis. Adult female mosquitoes were injected with polystyrene beads and re-injected 24 h later with fixed, fluorescently labeled *E. coli* or *S. aureus*. Microscopic observations showed that the capacity of hemocytes to engulf bacteria was drastically reduced in bead-injected compared to control mock-treated mosquitoes (Figure S6A). However, this blockade of phagocytosis affected only mildly the survival of mosquitoes infected with live bacteria (Figure S6B), as compared to the effect observed with PGRPLC silencing. Furthermore, the effect was statistically significant only in *S. aureus* and not in *E. coli* infections, suggesting that phagocytosis of *E. coli* may not (or only partly) explain the PGRPLC-mediated resistance to infections with this bacterium.

### Silencing *PGRPLC* increases infection by malaria parasites

**Figure 4:**
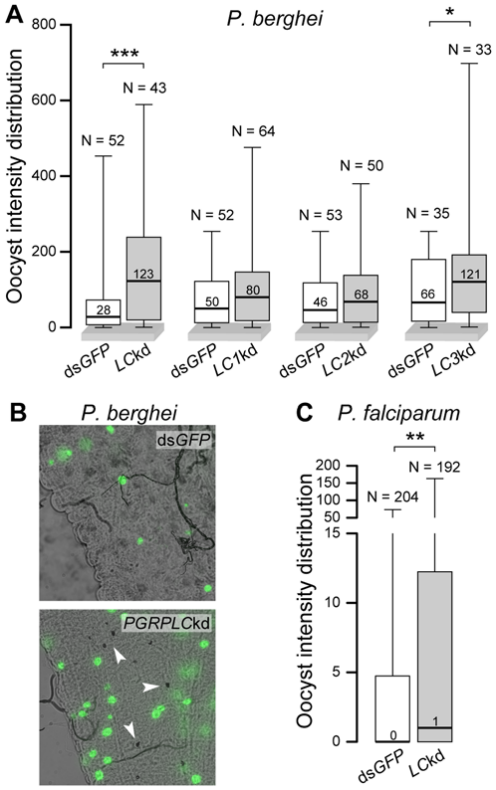
PGRPLC affects mosquito infections with *Plasmodium*. (A) Box plots of median numbers and distribution of oocyst intensities in *P. berghei*-infected ds*GFP*-treated (controls) or *PGRPLC* kd *A. gambiae* females. Independent controls were used for each of the entire *PGRPLC* gene (LC) and isoform-specific kds. Boxes show the distribution of 50% of the data and whiskers indicate the full range. N above each whisker indicates the numbers of mosquitoes. Results of Mann Whitney statistical tests are shown above each box plot: ***, P<0.001; *, P<0.05. (B) *P. berghei* 7-day-old oocysts and melanized ookinetes (arrowheads) in the midgut of *PGRPLC* kd and dsGFP-treated control *A. gambiae* females. (C) Box plots of median numbers and distribution of oocyst intensities of *P. falciparum* field isolates in ds*LacZ*-treated control or *PGRPLC* kd *A. gambiae*. **, P<0.01.

We assessed by RNAi the potential role of PGRPLC in mosquito infection with malaria parasites. *PGRPLC* kd and ds*GFP*-injected control mosquitoes were fed on mice infected with GFP-expressing *P. berghei* [20],[21] and their midguts were dissected 7 days later to determine the levels of infection. Silencing the entire *PGRPLC* gene resulted in a significant 4.4-fold increase of the median oocyst numbers relative to ds*GFP*-injected controls (P<0.001; Figure 4A). A very significant (P<0.05) increase in the prevalence of melanized ookinetes was also detected in *PGRPLC* kd mosquitoes (Figure 4B). Silencing each of the three isoforms independently revealed a significant effect of *PGRPLC3* kd on the median oocyst numbers (P<0.05), the infection prevalence (P<0.05) and the melanized ookinete prevalence (P<0.001). *PGRPLC2* kd also had a significant (P<0.01) effect on the prevalence of melanized ookinetes.

These data prompted us to examine the effect of *PGRPLC* kd in *A. gambiae* infections with the human malaria parasite *Plasmodium falciparum* in experiments performed in a high malaria transmission locale in Cameroon. Blood samples donated by *P. falciparum* gametocyte carriers were used to membrane feed laboratory-reared mosquitoes injected with either *PGRPLC* or control *LacZ* dsRNA. Five independent infections were performed, each using blood from a different gametocyte carrier, and oocyst intensities were determined 8 days later. The pooled data revealed a statistically significant (P<0.005) increase of the median oocyst intensity in *PGRPLC* kd mosquitoes compared to controls (Figure 4C). The infection prevalence also increased from 41% to 52% (Fisher's exact test P<0.005). Melanized *P. falciparum* ookinetes were not detected.

### PGRPLC signaling controls proliferation of gut microbiota and intestinal bacterial infections

**Figure 5:**
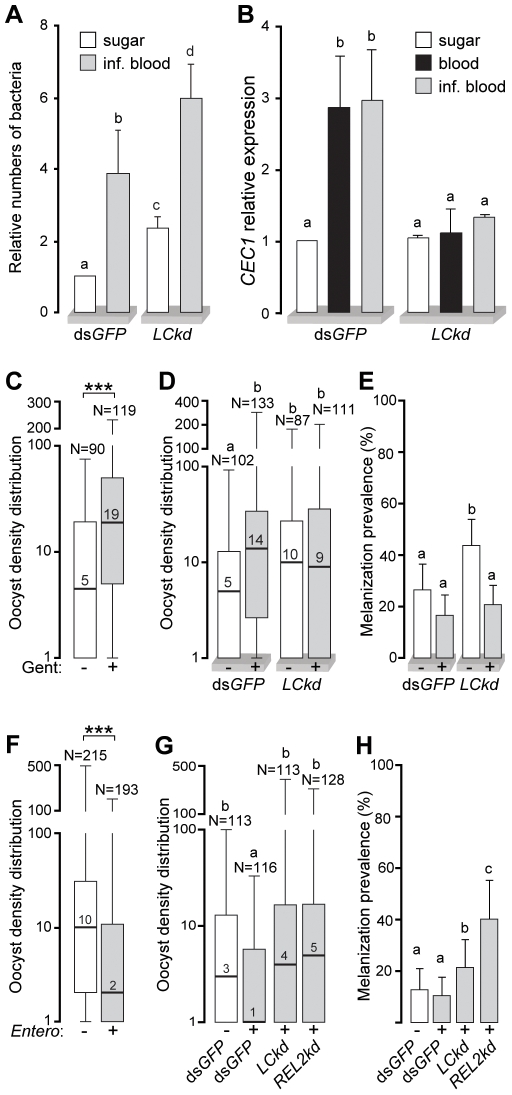
PGRPLC controls gut bacteria modulating *Plasmodium* infections. (A) Relative numbers of bacteria in ds*GFP*-treated control or *PGRPLC* kd *A. gambiae* females, fed on sugar or *P. berghei*-infected mice 24 h before sampling. Quantification was performed by quantitative genomic PCR of a conserved bacterial 16S rDNA fragment and referenced to sugar-fed controls. Error bars indicate standard errors. Comparisons between all the samples were performed using the Student's t-test and the results are shown as letters above each bar. Same letters indicate no significant difference, while different letters indicate at least P<0.05. In this graph all P values were <0.001. (B) *CEC1* relative expression in dsGFP-treated control and *PGRPLC* kd *A. gambiae* females fed on sugar, naïve blood or *P. berghei*-infected blood. Expression in sugar-fed controls is used as a reference. As in (A) different letters above each bar indicate Student's t-test P value<0.001. (C) Median numbers and distribution of *P. berghei* oocyst intensities in Gentamycin-treated (+) and untreated (−) mosquitoes. Boxes include 50% of the data and whiskers indicate the range in a log_10_-transformed scale. Median is shown with the bar and number within each box. ***, P<0.001 of Mann Whitney test. (D) Median numbers and distribution of *P. berghei* oocyst intensities in Gentamycin-treated (+) and untreated (−) *PGRPLC* kd and ds*GFP*-treated controls shown in a log_10_-transformed scale. As above, different letters above each dataset indicate statistically significant differences: P<0.001 for ds*GFP*(−)/ds*GFP*(+) and P<0.01 for ds*GFP*(−)/*LC*kd(−) and ds*GFP*(−)/*LC*kd(+). (E) Prevalence of melanized ookinetes in the mosquitoes presented in (D). Statistical analysis was performed with the Fisher's exact test: ds*GFP*(−)/*LC*kd(−), P<0.05; ds*GFP*(+)/*LC*kd(−), P<0.0001; *LC*kd(−)/*LC*kd(+), P<0.001. (F) Median numbers and distribution of *P. berghei* oocyst intensities in *Enterobacter*-infected (+) and non-infected (−) mosquitoes. Note that the y-axis is log_10_-transformed. (G) Median numbers and distribution of *P. berghei* oocyst intensities in *Enterobacter*-infected (+) and non-infected (−) ds*GFP*-treated mosquitoes, and in *Enterobacter*-infected *PGRPLC* kd and REL2 kd mosquitoes. Different letters above each dataset indicate statistically significant differences as follows: P<0.005 for ds*GFP*(−)/ds*GFP*(+) and P<0.001 for ds*GFP*(+)/*LC*kd(+) and ds*GFP*(−)/*LC*kd(+). (H) Prevalence of melanized ookinetes in the mosquitoes presented in (G). ds*GFP*(−)/*LC*kd(+), P<0.05; ds*GFP*(−)/*REL2*kd(−), P<0.0001; ds*GFP*(+)/*LC*kd(+), P<0.0001; ds*GFP*(+)/*REL2*kd(+), P<0.05; *LC*kd(+)/*REL2*kd(+), P<0.005.

Bacterial populations are common in the mosquito gut, and their size increases rapidly and substantially after a bloodmeal [22]. We examined whether PGRPLC signals against microbiota or opportunistic bacterial infections, which may interfere with mosquito infections with *Plasmodium*. *PGRPLC* kd and control ds*GFP*-treated 5-day-old adult females were sampled either after continuous sugar feeding or 24 h after blood feeding on mice infected with the rodent malaria parasite, *P. berghei*. DNA was extracted from surface-sterilized mosquitoes and used in quantitative genomic PCR reactions to assess the abundance of bacterial 16S ribosomal DNA (rDNA). Oligonucleotide primers were selected to match highly conserved regions of prokaryotic 16S rDNA [23], ensuring detection and quantification of most bacteria populations. Silencing *PGRPLC* led to a significant (P<0.001) 2-fold increase of the bacterial load in sugar-fed mosquitoes (Figure 5A). Following a bloodmeal, the increase of bacteria was approximately 4-fold in control and 6-fold in *PGRPLC* kd mosquitoes. This increase coincided with complete absence (P<0.001) of *CEC1* upregulation in *PGRPLC* kd mosquitoes compared to the 3-fold induction in their respective dsGFP-injected controls (Figure 5B). *CEC1* transcript levels were similar between mosquitoes fed on *Plasmodium*-infected and uninfected blood.

To investigate whether the effect of PGRPLC on *Plasmodium* is related to the presence of the midgut microbiota, we treated adult mosquitoes for 5 consecutive days after hatching from the pupal stage with the antibiotic gentamycin and then allowed them to blood feed on *P. berghei*-infected mice. The results revealed a highly significant (P<0.001) 3-fold increase of the oocyst numbers that developed in the mosquito midguts (Figure 5C). We then performed the same experiment using *PGRPLC* kd and control ds*LacZ*-injected mosquitoes and counted the number of oocysts or melanized parasites 7 days post infection. The numbers of oocysts in the gentamycin treated or untreated *PGRPLC* kd mosquitoes were similar between them and with those in gentamycin-treated ds*LacZ*-injected controls (Figure 5D). These data further supported our hypothesis that the effect of *PGRPLC* on *Plasmodium* survival is directly related to the bacteria residing in the mosquito midgut. Furthermore, the number and prevalence of melanized parasites in *PGRPLC* kd mosquitoes dropped to control levels when bacteria were depleted, suggesting that the parasite melanization phenotype was also related to the presence of bacteria in the mosquito midgut.

Next we performed the reverse experiment by feeding adult mosquitoes with bacteria for 2 consecutive days before they were blood fed on *P. berghei*-infected mice. We used 3 different bacteria species, *S. aureus*, *E. coli* and *Enterobacter cloacae* (Gram−), all of which led to a significant decrease of the number of oocysts in the mosquito midguts compared to control (Figure S7). Infection with *E. cloacae* had the biggest impact (P<0.001) on the numbers of oocysts (Figure 5F). We combined oral mosquito infections with *E. cloacae* with silencing *PGRPLC* or *REL2* before infection with *P. berghei*. Both gene KDs reversed the effect of *E. cloacae* infection, leading to a significant increase of the oocyst numbers (Figure 5G). In addition, silencing *PGRPLC* and *REL2* substantially increased the numbers of melanized ookinetes, especially the latter (Figure 5H).

### All PGRPLC isoforms can potentially bind both DAP- and Lys-type PGN

Our data suggested that PGRPLC3 might play a key modulatory role in the defense against bacteria, which in turn modulates infections with *Plasmodium*. To investigate this, we built homology models of the three main PGRPLC isoforms based on the crystal structure of the *Drosophila* PGRP-LCx-TCT-LCa heterodimer complex and structural alignments between *Anopheles* and *Drosophila* PGRPLCs (Figure S8). *Dm*PGRP-LCx choice as template was further justified by the structural rigidity of the PGN binding cleft, as revealed in 3D-superpositions (data not shown).

None of the *Ag*PGRPLC isoforms exhibit the two-residue insertions (IN and DF; Figure S8) that occlude the TCT (found in *E. coli*) binding groove in *Dm*PGRP-LCa [10] making this isoform deficient for PGN binding [15]. We used the TCT position in the *Dm*PGRP-LCx-TCT-LCa complex as the initial docking position and modeled the ability of *Ag*PGRPLCs to bind TCT (Figure S9). Docked TCT formed an extensive network of interactions with residues lining the binding groove of all AgPGRPLC isoforms (Figure S9, Text S1, Table S2, Table S3 and Table S4). Most of these are identical between *Ag* and *Dm*PGRPLCs and some are isoform-specific. The Arg-mediated recognition of DAP [11],[12] is mediated by R82 of *Ag*PGRPLC1 and LC2 and R84 of *Ag*PGRPLC3. A polar interaction between Y56 of *Ag*PGRPLC1and O6 of TCT provides direct recognition of the 1,6-anhydro bond that is essential for immunostimulatory activity [14],[24].

We also examined the potential of *Ag*PGRPLCs to bind Lys-type PGN (found in *S. aureus*), using as template the human PGRP-Ia-MTP (muramyl tripeptide) complex [25],[26]. A two-residue pattern (NY/F) at the rim of the PGN binding cleft that is thought to mediate specificity to Lys-type PGN [27] exists in all *Ag*PGRPLCs. In all the *Ag*PGRPLC-MTP models, docked MTP is indeed cradled by a combination of contacts conserved in hPGRP-Ia-MTP (Figure S9, Text S1, Table S2, Table S3 and Table S4). These data suggested no difference in the potential of PGRPLC3 and PGRPLC1 or LC2 to bind DAP- or Lys-type PGN.

### PGRPLC3 cannot initiate TCT-induced dimerization

Crystal structures of *Dm*PGRP-LCx-TCT-LCa and *Dm*PGRP-LE-TCT-LE and functional studies [14],[24] have shown that monomeric PGN induces functional dimerization of *Dm*PGRPs. We modeled *Ag*PGRPLC dimerization based on the *Dm*PGRP-LCx-TCT-LCa heterodimer. *Ag*PGRPLC-TCT models were placed at the position of *Dm*PGRP-LCx-TCT in the complex (position /x) and additional *Ag*PGRPLC models were placed at the position of *Dm*PGRP-LCa (position /a). The local structure at the dimer interface was maintained to account for induced-fit upon dimerization [11].

**Figure 6:**
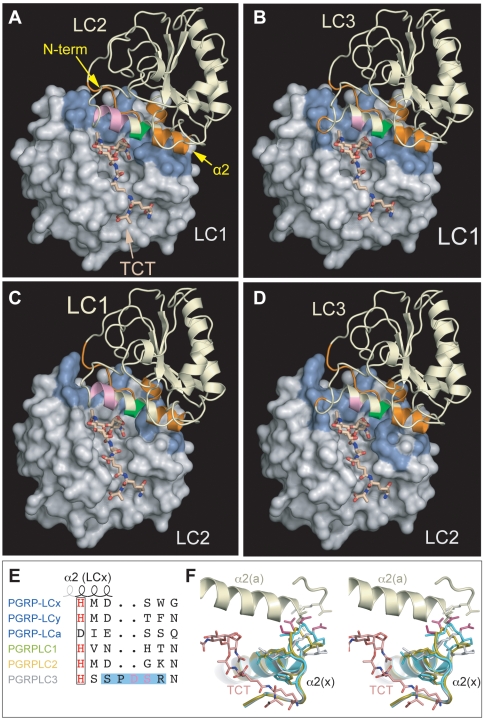
TCT-mediated hetero-dimerization of *Anopheles* PGRPLC isoforms. Heterodimer models of *Ag*PGRPLC1-TCT-LC2 (A), *Ag*PGRPLC1-TCT-LC3 (B), *Ag*PGRPLC2-TCT-LC1 (C) and *Ag*PGRPLC2-TCT-LC3 (D). PGRPLC/x molecules are shown in molecular surface models and PGRPLC/a in ribbon diagrams. The PGRPLC/a N-terminus and helix α2 that mediate dimerization are indicated, with monomer-interacting parts colored in orange, parts contacting both monomer and TCT in green and the TCT-interacting part in pink. Interface residues on the surface of PGRPLC/x are shown in blue. (E) Detail alignment of the PD-loop between *Ag* and *Dm* PGRPLCs, highlighting the modeled loop and clashing residues. (F) Stereo view of the putative dimer interface at the contact between helix α2/PD-loop of *Ag*PGRPLC3/x and helix α2 of *Ag*PGRPLC1/a (pale green). Three alternative *Ag*PGRPLC3/x models corresponding to different PD-loop modeling approaches are superimposed; in grey the model from MODELLER, in gold the average model structure from ARIA; and in turquoise the Robetta model. PD-loop Residues D61 and S62 (magenta), which clash severely with helix a2 in the three models, and the anchor, TCT-interacting residues R63 and F65, are shown in sticks.

The predicted interactions between *Ag*PGRPLC1/x or LC2/x and LC1/2/3/a revealed a pattern of contacts at the dimer interface consistent with the *Dm*PGRPLCx-TCT-LCa complex, no steric clashes and good stereochemistry. Molecular contact analysis suggested that binary complexes can be formed between *Ag*PGRPLC1 or LC2 at position /x and any of the three *Ag*PGRPLCs at position /a (Figure 6A–D and Text S1).

When attempting to place PGN-liganded *Ag*PGRPLC3 at position /x, we noticed a unique PD-insert immediately after helix α2 at position 60 of this isoform, which lies at a tight junction with helix α2 of *Ag*PGRPLCs/a (Figure 6E). As proline residues are helix terminators, the length of helix α2 is likely identical with *Dm*PGRP-LCx and the PD-insert is part of the PD-loop connecting the α2 and β2 helices. To explore the PD-loop conformation we used a conserved aromatic residue at position 65 of the α2/β2 loop (F65 of *Ag*PGRPLC3), which is packed firmly with DAP- or Lys-type PGN in all liganded structures, as the anchor point and specifically modeled the PD flanking sequence S59-PDSR-N64 using MODELLER [28]. In the resulting structure, R63 occupied a conserved binding position to TCT or Lys-PGN, equivalent to K61 in LC2/x (Text S1 and Table S3). Additionally, we used a simulated annealing protocol to allow for broad sampling of conformational space with accelerated energy barrier crossing, which produced an ensemble of ten lower-energy loop conformers (Figure 6F). While the PD-loop adopted a more relaxed conformation compared to the MODELLER structure, R63 and F65 were positioned in similar orientations, corroborating PGN-binding.

We sampled alternative and wider conformational space for the PD-loop using Robetta [29], a method that combines *de novo* and template-based protocols. Of the three resulting models, one showed PD-loop conformation similar to that of the simulated annealing and consistent with PGN binding (Figure 6F); the other two were inconsistent with PGN binding due to steric bumps or deformities in the L-shaped PGN-binding floor (data not shown). However, when the resulting *Ag*PGRPLC3/x-PGN models were positioned in heterodimers with LC1/a or LC2/a, D61 and S62 of the PD-loop clashed severely with residues at the inter-helical interface in the beginning of α2 helix/a, mainly at positions 42 and 44. These clashes could not be relieved by manual adjustment or energy minimization as they involved main-chain and C_β_ atoms, suggesting that AgPGRPLC3/x heterodimers are highly unlikely.

## Discussion

Recognition of conserved microbial structures is essential for activation of insect innate immunity. Sensing bacterial cell wall PGN by *Drosophila* PGRPs triggers one of two signaling pathways. Circulating PGRP-SA and PGRP-SD sense primarily Lys-type PGN that is mostly found in Gram+ bacteria, activating the Toll pathway that culminates with nuclear translocation of Dif or Dorsal. DAP-type PGN found mainly in Gram− bacteria and bacilli is principally sensed by the transmembrane PGRP-LC. This binding induces PGRP-LC dimerization and downstream initiation of Imd signaling that triggers activation and nuclear translocation of the Rel/NF-κB transcription factor Relish.

The *A. gambiae* ortholog of Relish, REL2, is required for resistance to infections with bacteria, both the Gram− *E. coli* and the Gram+ *S. aureus* [30]. Acting in concert with the IMD adaptor protein, REL2 confers resistance to *S. aureus* infections, but resistance to *E. coli* infections appears to be IMD-independent. Here we show that PGRPLC is essential in both these reactions, possibly acting as a common recognition receptor of these two branches of the REL2 antibacterial pathway. The first pathway resembles the conventional Imd pathway of *Drosophila*, consisting of PGRPLC, IMD and REL2-F, a form of REL2 that has an inhibitory ankyrin domain and a death domain. The second pathway appears to involve PGRPLC and REL2-S, a form of REL2 that lacks the ankyrin and death domains. The latter resembles a pathway proposed by Kaneko and colleagues [5], where IMD is dispensable for Imd pathway activation in response to DAP-type PGN. It has been speculated that an unknown factor interacts with an intracellular domain of PGRPLC other than the IMD interacting domain. Henceforth we will distinguish between these two REL2 pathways as IMD-dependent and IMD-independent, respectively.

Research in *Drosophila* has mainly used infection-dependent transcriptional induction of AMPs as diagnostic readout for defining pathways. Here, using survival of infected insects as the readout, we demonstrate the importance of *Drosophila* PGRP-LC in the defense against *S. aureus*, especially in the first days after infection; thereafter PGRP-SA becomes indispensable. These data indicate that the two pathways may act simultaneously and/or consecutively to maximize effectiveness: PGRP-LC and its downstream Imd signaling could effectively control low *S. aureus* infections, but persisting infections require PGRP-SA and Toll pathway responses. Vice versa, *Drosophila* PGRP-SA appears to also play a role in the defense against *E. coli* infections, but PGRP-LC is the principal receptor. This direct comparison of *Drosophila* and *Anopheles* indicates a putative divergence in PGRP-mediated antibacterial defenses between these two insects, as the mosquito PGRPLC seems to be the main receptor for both *E. coli* and *S. aureus*. This is consistent with our working hypothesis that adaptation of the evolutionary conserved signaling pathways is enabled by differences in recognition [31].

The *Anopheles* PGRPLC-mediated response to *E. coli* may not be the only defense against this bacterium. In contrast to the response to *S. aureus*, early transcriptional induction of AMPs in response to *E. coli* infections is not regulated by PGRPLC. An as yet unidentified PGRPLC-independent pathway may mediate this early response, but we cannot exclude that AMPs other than those tested here are induced via the IMD-independent REL2 pathway. The possibility that the PGRPLC requirement in *E. coli* infections relates to phagocytosis is unlikely, as blockade of phagocytosis appears not to have an effect equivalent to that of PGRPLC on mosquito survival following infections with this bacterium; however, further research is needed before this possibility can be entirely ruled out.

The mosquito gut is habitat to large and diverse microbial communities. Several species of bacteria have been identified in the gut of field-collected anophelines, mostly Gram− proteobacteria and enterobacteria [32],[33],[34]. Our data reveal that PGRPLC signaling controls the size of symbiotic bacteria populations and intestinal bacterial infections in laboratory reared *A. gambiae*. It also controls the proliferation of gut bacteria after a mosquito bloodmeal, which can be as drastic as several thousand folds [22] and coincides with robust PGRPLC-mediated AMP induction.

The importance of this reaction extends beyond mechanistic understanding of the mosquito immune system. *Anopheles* mosquitoes are vectors of *Plasmodium* parasites that cause malaria, a disease that infects 300–500 million people annually killing over one million of them. Within 24 h after a mosquito bloodmeal, a period that coincides with the proliferation of gut bacteria, *Plasmodium* gametocytes ingested with the blood undergo sexual development and reproduction, and the resulting zygotes become motile ookinetes that cross the midgut epithelium. The PGRPLC response against proliferating bacteria appears to be responsible, at least in part, for the drastic drop in parasite infection intensities during that period.

Importantly, we demonstrate that PGRPLC controls the intensity of *A. gambiae* infections by *P. falciparum* gametocytes found in the blood of children in sub-Saharan Africa. This is the first time that a gene of the classical mosquito immune system is shown to have an effect on *P. falciparum* field isolates. A similar effect is detected in infections with the common laboratory model parasite, *P. berghei*. These findings lead us to postulate that the tripartite interaction between symbiotic bacteria, the mosquito immune system and invading *Plasmodium* parasites may play an important role in the mosquito susceptibility vs. resistance to infection with malaria parasites. Since the various mosquito habitats or laboratory rearing conditions can have a direct impact on the mosquito gut microbiota, we speculate that the mosquito responses to *Plasmodium* may vary between different settings.

The combination of genetic, molecular and structural modeling data revealed a history of convergent evolution between the fruit fly and mosquito PGRPLC. Like its *Drosophila* ortholog, *Anopheles PGRPLC* gene produces three main isoforms via alternative splicing. However, these isoforms have evolved independently in the two lineages, via exon re-duplications [16],[31]. All isoforms share a common first part of the PGRP ectodomain; the remainder is distinct and encoded by two isoform-specific exons, separated by an intron at identical relative positions. This modular architecture allows generation via alternative splicing of a PGRPLC2/3 hybrid domain isoform, which is expressed at low levels and thus may have a modulatory function or be expressed in specific cell types or conditions.

An intriguing finding is that isoforms exist as short and long versions that differ by an alternative 25-aa cassette, laying immediately after the transmembrane domain and before the PGRP domain, an observation also made independently by Lin and colleagues [17]. A similar 17-aa cassette exists in *Drosophila* PGRP-LC. Both cassettes encode flexible structures and could be envisaged as providing long “necks” to membrane-bound PGRPLCs and hence flexibility to interact with ligands inaccessible to the rigid short-necked isoforms, e.g. PGN polymers shed by Gram+ bacteria. An alternative hypothesis derives from a study showing *Drosophila* PGRP-LC down regulation in response to live bacteria and suggesting its proteolytic cleavage by bacterial proteases as a sensing mechanism [35]. We propose that this cassette may be targeted for cleavage by host or pathogen proteases, resulting in immune response activation, down-regulation or evasion.

Our data suggest that PGRPLC3 is the most important of the three main isoforms in the response against bacterial infections. PGRPLC3 is essential for resistance to *E. coli*, whereas PGRPLC1 and LC2 play less important roles. PGRPLC3 is also central in defense to *S. aureus*, as is PGRPLC1 albeit less important. These two isoforms have been previously implicated in *CEC1* expression in cultured cells [17]. The specificity of PGRPs to these two bacteria is thought to be determined largely by the third aminoacid in the peptide bridge connecting their PGN glycan strands: DAP in *E. coli* and Lys in *S. aureus*. *Drosophila* PGRP-LCx is shown to bind DAP-type PGN, while PGRP-LCa is deficient for PGN binding [10],[15]. Our structural models reveal a potential of all *Anopheles* PGRPLC isoforms to bind both DAP- and Lys-type PGNs. These data suggest a remarkable structural flexibility of PGRPLCs in PGN recognition and provide mechanistic support to genetic data reported herein and in *Drosophila* studies, where PGRP-LC mediates resistance to Lys-type PGN Gram+ bacteria [36]. However, they do not explain the observations that *Anopheles* PGRPLC3 is the most important isoform in antibacterial defense.

Our PGRPLC dimerization models have provided important insights into this putatively unique role of PGRPLC3. Molecular contact analysis suggests that binary complexes, such as those formed between *Drosophila* PGRP-LCx and LCa upon TCT binding on the former [11], can exist between *Ag*PGRPLC1 or LC2 at the /x position and any of the three *Ag*PGRPLCs at the /a position. However, *Ag*PGRPLC3 cannot initiate dimerization with other isoforms, as a two-residue insertion obstructs its binding surface. Moreover, unspliced *PGRPLC3* transcripts are never detected, as opposed to all other splice variants, and PGRPLC3 is the dominant isoform in EST and cDNA sequences. These data could indicate a unique dual role of *Ag*PGRPLC3 (Figure S10): (a) it locks PGRPLC complexes induced by monomeric PGN binding on the other two isoforms in binary or oligomeric modes, preventing the formation of multimers and adopting a role similar to *Dm*PGRP-LCa; (b) it down regulates the response by removing from the environment monomeric PGN, since it cannot initiate formation of immunostimulatory dimers. The former role would maximize the response during high PGN concentrations, whereas the latter role would serve in dampening the immune signal in low PGN concentrations. This may justify the absence of PGRPLC-mediated induction of AMPs in early stages of *E. coli* infection and could also suggest a mechanism of immune tolerance to symbiotic bacteria.

## Materials and Methods

### Ethics statement

Research involving humans was approved by the WHO and Cameroon National Ethics Committees. Collection and analysis of data was conducted anonymously. Informed written consent forms were collected. Research involving animals was approved by the United Kingdom Home Office and Imperial College Review Board.

### *Anopheles*, *Drosophila* and *Plasmodium*

The *A. gambiae* G3 and N'gousso strains were reared as described in [37]. N'gousso is a laboratory-strain colonized in 2006 from field mosquitoes collected around Yaoundé, Cameroon. The *D. melanogaster white* mutant strain was obtained from Blades Biological Ltd, UK and the *PGRP-SA* (semmelweiss) [1] and *PGRP-LC*/TM6B [3] mutant strains were a kind gift of J. Royet. *P. berghei* was passaged through CD1 or Balb/C mice and mosquito infections were performed using standard procedures [38].

### RNA isolation and cDNA synthesis

Total RNA was isolated from 10 female mosquitoes using TRIzol (Invitrogen) and treated with DNaseI. First strand cDNA synthesis was performed with 5 µg total RNA, using oligo-d(T) primers (Invitrogen) and Superscript Reverse Transcriptase II (Invitrogen) according to the manufacturer's instructions.

### Quantitative real time RT-PCR

Amplifications were performed with SYBR Green PCR mastermix and analyzed using the ABI PRISM 7700 sequence detection system following the manufacturer's instructions. Expression levels were calculated by the relative standard curve method, as described in Technical Bulletin #2 of the ABI Prism 7700 Manual (Applied Biosystems), using S7 as endogenous control.

### DsRNA production and injection

Oligonucleotide primers flanked by T7 sequence (GAATTAATACGACTCACTATAGGG) were utilized in genomic PCR reactions for production of dsRNA. PCR products were cleaned up with the QIAquick PCR Purification kit (QIAGEN) and verified by sequencing. DsRNA was synthesized with the MEGAscript T7 Kit (Ambion) treated with DNaseI and cleaned up with the RNeasy kit (QIAGEN). Its concentration was adjusted to 3 µg/µl and 69 nl were injected into the dorsal side of the insect thorax as described [39]. DsRNA for the whole *PGRPLC* gene KD was produced by subcloning the EST clone 4A3B-AAA-E-04 [40] into the plasmid vector pLL10 [41].

### Bacteria cultures and infections

*E. coli* and *S. aureus* were cultured to 0.7 OD_600_, pelleted, washed and resuspended in PBS. Final OD_600_ for mosquito injections were 0.01 for *E. coli* and 0.4 for *S. aureus*, and for fruit flies 0.1 for *E. coli* and 0.01 for *S. aureus*. 69 nl of these or PBS for controls were injected into the insect thorax with a nano-injector (Nanoject, Drummond). Dead insects were counted daily and removed. At least three independent experiments were performed, each carried out with more than 50 female individuals.

For oral bacterial infections, *E. cloacae*, *S. aureus* and *E. coli* were cultured overnight in LB medium, washed twice in PBS, and re-suspended in 1% sucrose solution. The suspension was placed on cotton pads on which newly emerged mosquitoes were allowed to feed for 48 h.

### Antibiotics treatment

Freshly emerged mosquitoes were fed for 3 days with gentamycin (25 µg/ml) diluted in water and placed on a cotton pad. Sugar cubes were used as a sugar source. Fresh antibiotics solution was provided every 12 h. Mosquitoes remained on antibiotics for 2 more days before being infected with the *P. berghei* parasite as described above. For gene silencing, dsRNA was injected into mosquitoes 24 h after the beginning of the antibiotics treatment.

### Phagocytosis assay

A modified protocol from [42] was used. Red amine conjugated polystyrene beads (0.2 µm diameter (Molecular Probes) were washed twice in PBS and resuspended in PBS in the original volume. Adult females were injected with 100 nl into the thorax using a nano-injector (Nanoject, Drummond). One day later *E. coli* or *S. aureus* were prepared at OD_600_ = 1 and 69 nl were injected in each mosquito. The experiment was repeated three times and the percent survival rates were averaged and statistically analyzed using the Log rank (Mandel-Cox) and the Gehan-Breslow-Wilcoxon tests.

### Quantification of bacterial DNA

Five adult female mosquitoes were surface-sterilized in 70% ethanol for 5 min and rinsed three times in sterile saline (0.9% NaCl solution) as described [22]. Genomic DNA was extracted using the QIAamp DNA Mini Kit (QIAGEN) and quantified by real-time PCR using the BSF340/19 and BSF806/26 oligonucleotide primers to the bacterial rDNA (Table S1), the SYBR Green PCR mastermix and the ABI PRISM 7700 sequence detection system following the manufacturer's instructions. The *A. gambiae* S7 gene was used as a reference.

### *Plasmodium* infections

Mosquitoes were fed at 21°C for 15 min on TO mice infected with GFP-expressing *P. berghei* [21]. Midguts were dissected 10 days later, fixed for 45 min in 4% formaldehyde and mounted on glass slides in Vectashield. Fluorescent and melanized parasites were counted under fluorescence microscope.

For *P. falciparum* infections, schoolchildren of Mfou (30 km east of Yaoundé) were screened for gametocyte presence by thick bloodsmears stained with Giemsa. Blood from children with >20 gametocytes/µl was drawn by venipuncture. The serum was separated by centrifugation and replaced by non-immune AB serum. Mosquitoes were fed for 30 min on a 38°C-warm blood feeder. Midguts dissected 8 days later were stained with 0.4% Mercurochrome and developing oocysts were counted.

### Statistical analysis

*Anopheles* and *Drosophila* survival after infections with bacteria were analyzed using the Log-rank (Mantel-Cox) and Gehan-Breslow-Wilcoxon tests of the statistical package Prism version 5.0 (GraphPad Software Inc.). Survival rates at the various time points were averaged between biological replicates after being transformed into percentages, and average survival curves were constructed and compared.

The median of *Plasmodium* infection densities were analyzed using the non-parametric Mann Whitney test of Prism version 5.0, and the prevalence of infection and melanized ookinetes were analyzed using the Fisher's exact test.

### Homology modeling and DAP/Lys-PGN docking

Homology models were constructed in SwissModel after structural alignment of PGRP domains with Tcoffee [43] followed by manual adjustments. PD-loop modeling and optimization was performed in MODELLER 9v1 using the Discrete Optimized Protein Energy method [44]. PD-loop prediction methods included: a template-based protocol in Robetta (http://robetta.bakerlab.org) which uses the *de novo* Rosetta fragment insertion method [45]; and an implementation of ARIA [46], applying a Cartesian MD and simulated annealing protocol with a high-temperature torsion angle dynamics (TAD) stage (2000 K, 36 ps), two cooling steps (2000 to 1000 K over 30 ps and 1000 to 50 K over 24 ps) and a final energy minimization (200 steps) of the MODELLER-derived PD-loop model while all other atomic positions were restrained. An ensemble of 100 loop conformers was calculated and 10 conformers with the lowest total energy were analyzed.

To model TCT binding, TCT was docked in the PGN-binding groove of *Ag*PGRP models according to its binding position in PGRP-LCx-TCT-LCa. TCT-protein interactions were optimized manually using O [47] and the Penultimate rotamer library [48] for putative side chain interactions. The conformation of identical TCT-interacting residues in *Ag*PGRP models and PGRP-LCx was not changed and the side-chain χ1 angles of non-identical residues were maintained when possible. TCT torsions were allowed only to relieved clashes and did not compromise predicted binding. Liganded models were subjected to 200 cycles of conjugate gradient energy minimization in CNSsolve1.2 [49]. Similar steps were followed to model Lys-PGN binding, using a complex structure of *h*PGRP-IαC with a Lys-type MTP [50]. Model quality and intermolecular clashes analysis were performed with PROCHECK [51] and MolProbity [52]. Ligand-protein interactions were analyzed with LIGPLOT [53]. Final models had good stereochemistry without residues in non-permissive areas of Ramachandran plots.

### Modeling heterodimerization

To obtain *Ag*PGRPLC/a models, the portions of helix α2 and N-term segment of LCa mediating contacts with LCx were modeled using SwissModel to map corresponding *Ag*PGRPLC sequences, which accounted for induced fit upon dimerization. Side-chain conformations of identical residues at the dimer interface were altered only if necessary for non-identical residues by manual manipulations in O. *Ag*PGRPLC/a models were positioned by superimposing backbone atoms of helix α2 and the N-term of *Ag*PGRPLC/x models onto the corresponding regions of LCa using LSQMAN [54]. Inter-monomer contact analysis was performed with LIGPLOT and MolProbity. Figures were generated with Pymol [55].

## Supporting Information

Text S1 AgPGRPLC-TCT and AgPGRPLC-Lys complexes, and TCT-mediated AgPGRPLC heterodimers.
(0.12 MB PDF)

Table S1 Oligonucleotide primers used in PCR and RT-PCR reactions.
(0.08 MB PDF)

Table S2 Interactions in AgPGRPLC1-MTP/TCT model structures.
(0.08 MB PDF)

Table S3 Interactions in AgPGRPLC2-MTP/TCT model structures.
(0.08 MB PDF)

Table S4 Interactions in AgPGRPLC3-MTP/TCT model structures.
(0.08 MB PDF)

Figure S1 Injection of bacterial suspensions but not with saline alone causes progressive mosquito mortality. Following the indicated injections in female mosquitoes, survival is recorded daily for 5 days. Mortality is minimal and slow after saline injection alone, but rapid and progressive after *S. aureus* or *E. coli* injections.
(0.23 MB TIF)

Figure S2 Oligonucleotide primers used in PGRPLC PCR and RT-PCR reactions. The relative position and orientation of primers on the PGRPLC genomic locus is shown. Numbers indicate the primer numbers presented in Table S1.
(0.21 MB TIF)

Figure S3 Amino acid alignment of *A. gambiae* and *D. melanogaster* PGRPLC main isoforms. The predicted Imd interaction region in Drosophila (yellow), RHIM-like motifs (blue), transmembrane domains, alternative cassettes (orange) and PGRP domains are shown. Intron positions are indicated with black arrowheads.
(0.80 MB TIF)

Figure S4 RT-PCR reveals the presence of various PGRPLC isoforms and a pool of unspliced transcripts. Each subpanel (A–J) features a different PCR primer combination and the corresponding gene model prediction. Grey bars above these models represent sequenced RT-PCR products shown on the right side of each panel.
(1.78 MB TIF)

Figure S5 Efficiency of PGRPLC isoform silencing in *A. gambiae*. Relative percent expression of each of the three main *PGRPLC* isoforms quantified by qRT-PCR in adult *A. gambiae* females silenced for the expression of entire *PGRPLC* gene or each of the three isoforms. A small increase of *LC1* transcript levels in *LC3* kd mosquitoes may suggest an effect of silencing on the pool of unspliced transcripts, resulting in upregulation of non-targeted transcripts.
(0.72 MB TIF)

Figure S6 Blocking phagocytosis does not have a major effect on mosquito survival. (A) Indicative microscopic images of hemocytes in *A. gambiae*injected with PBS (top) or Red amine conjugated polystyrene beads (bottom) and re-injected 24 h later with FITCconjugated *E. coli*. BF, bright field. (B) Percent survival of mosquitoes subjected to the same procedures as in (A) but injected with live instead of fixed *E. coli* or *S. aureus* bacteria. Mortality rates of bead-injected mosquitoes were marginally increased compared to control PBS injected and statistically significant (P<0.05) with the Log rank (Mandel-Cox) and the Gehan-Breslow-Wilcoxon tests in *S. aureus*, but not in *E. coli*, infections. The presented survival rates are the average of three independent biological replicates.
(1.72 MB TIF)

Figure S7 Effect of mosquito midgut infections with different bacteria on *P. berghei* infection intensities. Median numbers and distribution of *P. berghei* oocyst intensities in *E. coli* and *S. aureus* infected and in control non-infected mosquitoes. Boxes include 50% of the data and whiskers indicate the range in a log10-transformed axis. Median is shown with the bar and number within each box. *, P<0.01; **, P<0.005; N, numbers of mosquitoes.
(0.72 MB TIF)

Figure S8 Structure-based multiple alignment of *A. gambiae* (red) and *D. melanogaster* (blue) PGRP domains. The secondary structure of PGRP-LCx in the *Dm*PGRP-LCx-TCT-LCa complex is shown above sequences. Asterisks next to PGRP names indicate predicted or experimentally validated amidase activity. Known preferences of PGRPs to Lys and DAP-type PGN are noted. Purple triangles indicate residues implicated in zinc coordination and stars mark catalytically important residues in T7 lysozyme. Brown triangles indicate residues of *Dm*PGRP-LCx that interact with TCT in *Dm*PGRP-LCx-TCT-LCa complex. Green and blue triangles show residues that interact with MTP and shape the PGN binding pocket, respectively, in human PGRP-IaC. Green lines indicate cysteines involved in disulfide bridges.
(1.36 MB TIF)

Figure S9 Surface view of *Ag*PGRPLC models in complex with Lys- and DAP-type PGN. The Lys-type muramyl peptide MurNac-L-Ala-D-isoGln-L-Lys (A, B, C) and the DAP-type PGN fragment TCT (D, E, F) are shown in sticks docked to their predicted binding positions in the PGN-binding grooves of *Ag*PGRPLC1, LC2 and LC3, respectively. Secondary-structure elements are visible under semi-transparent surfaces.
(6.58 MB TIF)

Figure S10 Schematic hypothetical model for the role of PGRPLC3 in modulating immune signaling. (A) Low amounts of PGN are sequestered on the cell surface by the abundant PGRPLC3 that cannot induce dimerization thus dampening the signal. (B) Other PGRPLC isoforms are also engaged in binding PGN that is in high concentrations during high infections and initiate dimerization with PGRPLC3, which thereby locks these isoforms in immunostimulatory dimeric complexes.
(0.82 MB TIF)
