# Supplementary material for: Anopheles gambiae PGRPLC-Mediated Defense against Bacteria Modulates Infections with Malaria Parasites
Source: PLoS Pathog. 2009 Aug 7;5(8):e1000542. doi: 10.1371/journal.ppat.1000542 (PMC2715215; doi:10.1371/journal.ppat.1000542)
Supplement: Text S1 — AgPGRPLC-TCT and AgPGRPLC-Lys complexes, and TCT-mediated AgPGRPLC heterodimers. (0.12 MB PDF) [file ppat.1000542.s001.pdf]

Versatile PGRPLC-mediated modulation of *Anopheles gambiae* antibacterial defense and infection with malaria parasites

Stephan Meister, Bogos Agianian, Fanny Turlure, Angela Relogio, Isabelle Morlais, Fotis C. Kafatos, and George K. Christophides

Supporting data

*Ag*PGRPLC-TCT complexes

In the *Ag*PGRPLC1-TCT model, the ligand conformation remained unchanged. In *Ag*PGRPLC2-TCT small rotations of torsion angles between L-Ala and D-Glu were necessary to relieve steric clashes between the carboxyl group of D-Glu and P92. In *Ag*PGRPLC1 and LC3 however, Pro92 is replaced by a Lys, which permits placement without a bump. Most conserved interactions are maintained in *Ag*PGRPLC2-TCT interactions are maintained while the repositioned carboxyl group of D-Glu approaches D147 (which is equivalent to K147 and A148 in *Ag*PGRPLC1 and LC3, respectively) at H-bonding distance. In the *Ag*PGRPLC3-TCT complex, a small rotation around the glycosidicGlcNAc/MurNAc (anhydro) bond elevates the pyranose ring of GlcNAc by almost 0.7 Å away from the glycan-binding cleft. This motion was a result of non-permissive van der Waals contacts with the phenyl group of F150. This unique Phe replaces a conserved Thr residue (T149 in *Ag*PGRPLC1 and LC2) whose Oγ atom forms a water-mediated H-bond with ring-O5 atom of GlcNAc. However, the lost H-bond is replaced by π-stacking between F150 and the ring of GlcNAc (Table S4), a favorable interaction in protein-sugar complexes.

Compared to the *Dm*PGRP-LCx-TCT complex, three hydrogen bonds are lost in *Ag*PGRPLC1-TCT and *Ag*PGRPLC2-TCT; for *Ag*PGRPLC1 due to replacement of residues W, H and S with T, A and K at positions 61, 143 and 147, respectively and for *Ag*PGRPLC2 due to replacement of residues H, K with residues S, P at positions 143 and 92, respectively and torsional shifts in TCT. Two such bonds are lost in the *Ag*PGRPLC3-TCT complex (residue F150 and A148 in place of T and S in LCx). One additional H-bond is gained in PGRPLC1-TCT and PGRPLC2-TCT, whereby atoms OH of Y56 and N of G93 interact with O6 of MurNAc(anhydro) and O of D-Ala, respectively. The *Ag*PGRPLC3-TCT interaction is considerably more hydrophobic than the one observed in *Ag*PGRPLC1-TCT and *Ag*PGRPLC2-TCT (Table S4).

*Ag*PGRPLC-Lys complexes

Specific hydrogen bonding of amine N2 atom of D-*iso*Gln via residues D147 and H144of *Ag*PGRPLC2 and *Ag*PGRPLC3, respectively (Tables S3 and S4) is observed only in the hPGRP-Ia-MTP complex. Conversely, a predicted recognition of 2-acetamide N atom of MurNAc in the *Ag*PGRPLC1-MTP model (Table S2) is seen only in the hPGRP-Ia-MPP complex. This mixed pattern is also observed for a number of intimate van der Waals contacts between our models and MTP. Apart from conserved interactions, a new water-mediated contact to atom O4 of MurNAc is produced in *Ag*PGRPLC1-MTP and *Ag*PGRPLC2-MTP, which is missing in *Ag*PGRPLC3-MTP (Table S2-S4) assuming that W5, a structural water molecules observed in *Dm*PGRPLCx-

TCT-LCa [1] is conserved.

TCT-mediated AgPGRPLC heterodimers

Three residues in helix α2 of LCa (Figure 5) make key hydrophobic contacts to LCx while Arg51 at the middle of the helix interacts specifically with both LCx and MurNAc(anhydro) of TCT [1]. In putative *Ag*PGRPLC1-LC2 and LC1-LC3 dimers, the hydrophobic interactions are conserved (mediated by residues 44, 47 and 48). In LC2-LC1 and LC2-LC3 the interaction via residue 47 is missing, however, a number of new hydrophobic contacts are predicted (four for LC2-LC1 and three for LC2-LC3) via residues in both helix α2 and the N-terminal clamp. Arg51 is structurally conserved in *Ag*PGRPLC2 while it is replaced by a Gln in *Ag*PGRPLC1 and a Lys in *Ag*PGRPLC3. Both these substituted residues interact with adjacent monomer and TCT similarly to Arg51 (see below). Interestingly, a point insertion at position 110 of *Ag*PGRPLC1 and LC2 (Figure S7) is predicted to change the local conformation of loop β4/α3 in a way that allows interaction of T109/x and E108/x to R17/a in complexes LC1-LC2 and LC1-LC3, respectively, while similarly in LC2-LC1 and LC2-LC3, Y109/x would stack with E18/a. Additionally, due to the PD-insertion after helix α2 in LC3 an extra van der Waals contact between P60 of LC3 and I150 (LC1) or V150 (LC2) may be formed in complexes LC1-LC3 and LC2-LC3 (Figure 6 B, D).

An important contact in *Dm*PGRPLCx-TCT-LCa, which is also observed in *Dm*PGRPLE-TCT-LE, is a buried hydrogen bond between a Ser at the end of helix α2 in PGRP-LCx (S391) with a backbone carbonyl oxygen at the beginning of the same helix in PGRP-LCa. In *Ag*PGRPLC1 the equivalent residue is a His (H60) which after energy minimization (see Experimental Procedures) adopts a non-hydrogen bonding orientation. However, with minor changes in φ/ψ angles we could manually reposition H60 so that its $N_{\delta 1}$ atom is within H-bonding distance to the carbonyl. Therefore it cannot be excluded that this specific H-bond is formed. Nonetheless, H60 is positioned in intimate van der Waals contact with R47 and M47 in *Ag*PGRPLC1-LC2 and *Ag*PGRPLC1-LC3, respectively. In *Ag*PGRPLC2 the Ser is replaced by a Glycine and the hydrogen bond cannot be formed.

Another crucial interaction in *Dm*PGRPLCx-TCT-LCa, is mediated by a hydrophobic leucine residue at the N-terminal clamp of LCa which locks into a hydrophobic pocket formed by TCT and LCx. This essential Leu is conserved in all *Ag*PGRPLCs and docks into similar hydrophobic pockets. Moreover, in *Ag*PGRPLC2-LC1 and LC2-LC3, a new specific H-bond between Asp59 (LC2/x) and Thr44 (LC1/a) or Gln44 (LC3/a) is predicted. The same H-bond is also predicted in complex LC1-LC3, between N59 (LC1) and T44 (LC3). Apart from being hydrogen bonded, residues 59 and 44 interact also via van der Waals contacts in all possible heterodimers with *Ag*PGRPLC1/x and LC2/x.

*Dm*PGRP-TCT dimer structures revealed the key interactions involved in the recognition of the disaccharide moiety of TCT by molecules/a. These interactions are essential for TCT-induced dimerization [2], as manifested by the requirement of the saccharide moiety of PGN for stimulatory activity [3]. These contacts are mediated by direct hydrogen bonding of the 3-OH and 2-acetamide NH of GlcNAc to a conserved glutamate (E409 in LCa and E231 in LE), while the diozolane ring and atom O5 of MurNAc(anhydro) interact with the guanidine plane of a conserved arginine (R401 in LCa and R223 in LE). In *Dm*PGRPLCx-TCT-LCa, the 2-acetamide N atom makes two additional water-mediated H-bonds to residues Q364 and T405 of LCa. These polar bonds are not observed in *Dm*PGRPLE-TCT-LE where they are substituted by hydrophobic packing with residue C227, exacerbating the remarkable plasticity in PGN recognition as we also predict in our dimer models. *Ag*PGRPLC1/a, LC2/a and LC3/a recognized

MurNAc similarly to LCa and LE via the side chains of Q51, R51 and K51, respectively. The interaction with GlcNAc however showed variation because N59 of LC1, D59 of LC2 and S59 of LC3, replace the conserved glutamate, while the equivalent of residue T405 in LCa is R55 in LC1 and E55 in LC2 and LC3. Thus, in complex LC1-LC2, 3-OH and 2-acetamide NH are hydrogen bonded to carboxyl oxygens of D59 and E55 of LC2/a. In LC2-LC1 however, the same GlcNAc atoms form two respective water-mediated hydrogen bonds with N59 and Q14 (equivalent to Q364 of LCa) of LC1/a, assuming spatial conservation of a unique TCT-LCa bridging water molecule [1]. Remarkably, in the same complex, R55 could hydrogen bond to 4-OH of GlcNAc, a previously unseen connection. Finally, in LC1/LC2-LC3, the above E55-2-acetamide NH hydrogen bond is maintained but recognition of 3-OH appears to be lost.

It is also noted that apart from the above specific water-mediated H-bonds several other water bridges were maintained in *Ag*PGRP heterodimer models compared to *Dm*PGRP-TCT dimer structures (not shown); however, these were not explicitly taken into account in our analysis or calculations.

References

1. Chang CI, Chelliah Y, Borek D, Mengin-Lecreulx D, Deisenhofer J (2006) Structure of tracheal cytotoxin in complex with a heterodimeric pattern-recognition receptor. Science 311: 1761-1764.
2. Lim JH, Kim MS, Kim HE, Yano T, Oshima Y, et al. (2006) Structural basis for preferential recognition of diaminopimelic acid-type peptidoglycan by a subset of peptidoglycan recognition proteins. J Biol Chem 281: 8286-8295.
3. Stenbak CR, Ryu JH, Leulier F, Pili-Floury S, Parquet C, et al. (2004) Peptidoglycan molecular requirements allowing detection by the Drosophila immune deficiency pathway. J Immunol 173: 7339-7348.
