# Supplementary material for: Anopheles gambiae PGRPLC-Mediated Defense against Bacteria Modulates Infections with Malaria Parasites
Source: PLoS Pathog. 2009 Aug 7;5(8):e1000542. doi: 10.1371/journal.ppat.1000542 (PMC2715215; doi:10.1371/journal.ppat.1000542)
Supplement: Table S1 — Oligonucleotide primers used in PCR and RT-PCR reactions. (0.08 MB PDF) [file ppat.1000542.s002.pdf]

Table S1. Oligonucleotide primers used in PCR and RT-PCR reactions

Gene Name or PGRPLC primer	Forward (F) or Reverse (R)	Sequence	Product size or location info
PGRPS1	F	GGACGAGCCGGCCCAGGAATC	493 bp
	R	CAGCTTCGCGTACAGATAC	
PGRPLD	F	GACTCGGAGGATTGTCTGGA	281 bp
	R	CGGATCGACTCGGTGATAAA	
PGRPLB	F	CCATCCCGTACGTCATCATA	274 bp
	R	ATGTTCTTCGGTGGCAAATC	
PGRPLA1	F	CCGACATTCCAAGCAACTTT	280 bp
	R	ACCAGCCTAGCGTACAGCAT	
PGRPLA2	F	TGCTGATAACGCACATAGGC	124 bp
	R	TTGCTCGGTATGTCTTGCAG	
PGRPS2&3	F	ACAACTTCCTGGTCGGTGAG	
PGRPS2	R	TCACCTGTCACAATGGTCGT	533 bp
PGRPS3	R	CCCCACATTAAGCTACGTTTC	423 bp
PGRPLC1	F	AAAGTTGGAGCCCACACCAAA	234 bp
	R	CAAAAGCCTCAGCTGTTC	
PGRPLC2	F	ACCATTCCCGGTTACAATTC	273 bp
	R	CTCGTGATCGGGGCGCTCGTC	
PGRPLC3	F	AAAGGGTTCAACGTGGACAGC	282 bp
	R	TCGTCGGTTTGTGGTGTCGTT	
PGRPLC; qRT-PCR primers	F	CCGGTTAATAACGTCATCATTGC	
	R	TCACCGTAGTTGGTGTGGTT	119 bp
	R	CGTCGTAGTTTTTGCCATCC	118 bp
	R	TCGGGACTCGAATGAAACTC	107 bp
S7	F	GTGCGCGAGTTGGAGAAGA	78 bp
	R	ATCGGTTTGGGCAGAATGC	
GFP	F	TAATACGACTCACTATAGGGCAAGACACGTG CTGAAGTCAA	~450 bp

	R	TAATACGACTCACTATAGGGGCCTGAATTTA ACCAGGAACC	
BSF340/19 & BSF806/26	F	TCCTACGGGAGGCAGCAGT	
	R	GGACTACCAGGGTATCTAATCCTGTT	
CEC1	F	ACCAACCAACCACCAAACAAC	168 bp
	R	CTTCTCTGCTGCCTTGAACACT	
DEF1	F	GCCTTTGTGCCGCTCACT	61bp
	R	GCCTTACTGTTGCAGTAACCACC	
1	F	AGGAACCTACAGCTAGAAAATG	2nd exon
2	F	GTTAAGGTTGCCGTGGAAGC	2nd exon
3	F	CAACTCGTCGGACATCACGTT	2nd exon
4	R	ACCTGCCCCTTGATGTACGTT	2nd exon
5	R	ATCCTGGTAAATGTTCTTTATCAC	2nd exon
6	F	CACGCACCTGGCAATCTAGTCT	3rd exon
7	F	AACCCTTGATCAGCTTTATCG	3rd exon
8	R	TGGCACACAGGACAATCATCA	3rd exon
9	R	GGATCGATATGACCGCTACAA	3rd exon
10	F	CCAGATCCGAGACCGTTACGG	4th exon
11	F	AGACCGTTACGGTTAGTGACG	4th exon
12	F	CCGGTTAATAACGTCATCATTGC	4th exon
13	R	GTGGCAGTGTGAGCAATGA	4th exon
14	R	ACGGCATGCGGCCTGAG	4th/5th exon
15	R	TGCTTTGCATGCTTGCTGA	4th/5th exon
16	R	TACCTGATACATGCATTTCGTCTGA	4th/5th exon
17	F	CACAAAACACTTCTGACCTCTCC	4th intron
18	F	TCTGACCTCTCCCATGCAG	4th intron
19	F	AAACCACACCAACTACGGTGA	5th exon
20	R	TCACCGTAGTTGGTGTGGTT	5th exon
21	F	ACAACTTTTTGATCGGTGGTG	5th exon
22	F	AAAGTTGGAGCCCACACCAAA	5th exon

23	F	GCTTGCCAAGGAGTACAAGC	6th exon
24	R	CAAAAGCCTCAGCTGTTC	6th exon
25	R	AATGTGGCAAAAGCCTCAG	6th exon
26	R	AAATGTGGCAAAAGCCTCAG	6th exon
27	R	AAAATGTGGCAAAAGCCTCA	6th exon
28	F	GGGTGCGCTTAATTCAGGA	7th exon
29	F	ATGGATGGCAAAAACTACGAC	7th exon
30	R	CGTCGTAGTTTTTGCCATCC	7th exon
31	F	ACCATTCCCGGTTACAATTC	7th/8th exon
32	R	CTCCGAGGCATATATTCGGTA	8th exon
33	R	CAGTGCTTCGATTAGCCACTT	8th exon
34	R	CAAGCTGTGCAGTGCTTCGAT	8th exon
35	R	AAACTGGGGCAAGCTGTGCAG	8th exon
36	R	CTCGTGATCGGGGCGCTCGTC	8th exon
37	R	TCGGGACTCGAATGAAACTC	9th exon
38	F	TTTAGCGACATTGCGTATCA	9th exon
39	F	AAAGGGTTCAACGTGGACAGC	9th/10th exon
40	R	CCAGTGGGGCCAAGTTTTGATG	10th exon
41	R	TCGTCGGTTTGTGGTGTCGTT	10th exon
42	R	GGAAGTCATCAAGGACACTTGG	3' UTR
43	R	GGTTCTCTTTTTCCGGTTCA	3' UTR
