# Supplementary material for: Anopheles gambiae PGRPLC-Mediated Defense against Bacteria Modulates Infections with Malaria Parasites
Source: PLoS Pathog. 2009 Aug 7;5(8):e1000542. doi: 10.1371/journal.ppat.1000542 (PMC2715215; doi:10.1371/journal.ppat.1000542)
Supplement: Table S2 — Interactions in AgPGRPLC1-MTP/TCT model structures. (0.08 MB PDF) [file ppat.1000542.s003.pdf]

Table S2. Interactions in AgPGRPLC1-MTP/TCT model structures

Hydrogen bonds

PGRPLC1 atoms		Atoms of MTP		PGRPLC1 atoms		Atoms of TCT	
H57	Nε2	AMU	O7	H34	Nε2	GlcNAc	O7
T149	Oγ1		O4 (W5)[1]	T149	Oγ1		O5 (W5)[1]
T35	O		N2	Y56	Oη		O6 (anh)
Y68	Oη	L-Ala	O10	I150	N		O3
H90	O	D-*iso*Gln	N	T35	O	MurNAc	N2
K92	N		O	H57	Nε2		O7
N95	Nδ2		Oε1	Y68	Oη		O10
N95	Nδ2	Lys	O	H90	O	D-Glu	N
G88	O		O (W22)[1]	K147	Nζ		Oε1
				K92	N		Oε1
				N95	Nδ2		Oδ
				R82	Nη1	Meso-DAP	Oζ1
					Nη2		Oζ2
				G64	N		Oζ2
				G88	O	D-Ala	N (W22)[1]
				N93	N		O

Hydrophobic contacts

PGRPLC1 residues	Atoms of MTP		PGRPLC1 residues	Atoms of TCT	
T35	AMU	C8	A36	GlcNAc	C8
S151		C11	I150		C8
K147	D-*iso*Gln	C			C7
K92		Cβ	H34		C8
A89	Lys	Cα	S151	MurNAc	C11
		Cδ	H57	L-Ala	Cβ
Y63		Cβ	K147	D-Glu	Cε
		Cγ	H90		Cβ
		Cδ	A89		Cδ
		Cε	Y63	Meso-DAP	Cβ
					Cδ
			A89		Cβ
					Cα
			K92	D-Ala	Cα
					C

[1]Structural water molecules observed in *Dm*PGRP-LCx-TCT-LCa structure
