# Supplementary material for: Anopheles gambiae PGRPLC-Mediated Defense against Bacteria Modulates Infections with Malaria Parasites
Source: PLoS Pathog. 2009 Aug 7;5(8):e1000542. doi: 10.1371/journal.ppat.1000542 (PMC2715215; doi:10.1371/journal.ppat.1000542)
Supplement: Table S3 — Interactions in AgPGRPLC2-MTP/TCT model structures. (0.08 MB PDF) [file ppat.1000542.s004.pdf]

Table S3. Interactions in AgPGRPLC2-MTP/TCT model structures

Hydrogen bonds

PGRPLC2 atoms		Atoms of MTP		PGRPLC2 atoms		Atoms of TCT	
H57	Nε2	AMU	O7	H34	Nε2	GlcNAc	O7
T149	Oγ1		O4 (W5)[1]	T149	Oγ1		O5 (W5)[1]
T35	O		N2	V150	N		O3
Y68	Oη		O10	T35	O	MurNAc	N2
K61	Nζ	L-Ala	O (W74)[1]	H57	Nε2		O7
T90	O	D-*iso*Gln	N	K61	Nζ		O7 (W74)[1]
D147	Oδ2		N2	Y68	Oη		O10
N95	Nδ2		Oε1	K61		L-Ala	O (W74)[1]
N95	Nδ2	Lys	O	T90	O	D-Glu	N
G88	O		O (W22)[1]	D147	Oδ2		Oε2 (W190)[1]
				N95	Nδ2		Oδ
				R82	Nη1	Meso-DAP	Oζ1
					Nη2		Oζ2
				D64	N		Oζ2
				N95	Nδ2	D-Ala	O
				G93	N		O

Hydrophobic contacts

PGRPLC2 residues	Atoms of MTP		PGRPLC2 residues	Atoms of TCT	
T35	AMU	C8	A36	GlcNAc	C8
H57	L-Ala	Cβ	H34		C8
Y63	D-*iso*Gln	Cγ	V150		C7
P92		Cβ	H57	L-Ala	Cβ
		C	Y68		Cβ
Y63	Lys	Cα	T90		Cβ
		Cγ	P92	D-Glu	Cε
		Cε			Cβ
A89		Cα			Cγ
			Y63		Cγ
			A89		Cδ
			Y63	Meso-DAP	Cβ
			A89		Cα
			P92	D-Ala	C

[1]Structural water molecules observed in *Dm*PGRP-LCx-TCT-LCa structure
