# Supplementary material for: Anopheles gambiae PGRPLC-Mediated Defense against Bacteria Modulates Infections with Malaria Parasites
Source: PLoS Pathog. 2009 Aug 7;5(8):e1000542. doi: 10.1371/journal.ppat.1000542 (PMC2715215; doi:10.1371/journal.ppat.1000542)
Supplement: Table S4 — Interactions in AgPGRPLC3-MTP/TCT model structures. (0.08 MB PDF) [file ppat.1000542.s005.pdf]

Table S4. Interactions in AgPGRPLC3-MTP/TCT model structures

Hydrogen bonds

PGRPLC3 atoms		Atoms of MTP		PGRPLC3 atoms		Atoms of TCT	
H57	Nε2	AMU	O7	H34	Nε2	GlcNAc	O7
T35	O		N2	S152	N		O7
Y70	Oη		O10	E151	N		O3
R63	Nε/Nη2	L-Ala	O (W74)[1]	T35	O	MurNAc	N2
H92	O	D-*iso*Gln	N	H57	Nε2		O7
H144	Nε2		N2	R63	Nε/Nη2		O7 (W74)[1]
N97	Nδ2		Oε1	Y70	Oη		O10
N97	Nδ2	Lys	O	R63	Nη2	L-Ala	O (W74)[1]
G90	O		O (W22)[1]	H92	O	D-Glu	N
				H144	Nε2		Oε2
				N97	Nδ2		Oδ
				R84	Nη1	Meso-DAP	Oζ1
					Nη2		Oζ2
				S66	N		Oζ2
				N97	Nδ2	D-Ala	O
				G95	N		O

Hydrophobic contacts

PGRPLC3 residues	Atoms of MTP		PGRPLC3 residues	Atoms of TCT	
T35	AMU	C8	A36	GlcNAc	C8
S152		C11	H34		C8
F150	D-*iso*Gln	C	E151		C8
K94		Cβ			C7
A91		Cδ	F150		C4
A91	Lys	Cα			C5
F65		Cγ			C6
		Cδ	S152	MurNAc	C11
			H57	L-Ala	Cβ
			F150	D-Glu	Cε
			H92		Cβ
			A91		Cγ
					Cδ
			F65	Meso-DAP	Cβ
					Cδ
			A91		Cα
					Cβ
			S66		Cζ
			K94	D-Ala	Cα
					C

[1]Structural water molecules observed in *Dm*PGRP-LCx-TCT-LCa structure
