# Supplementary figures and images for: Anopheles gambiae PGRPLC-Mediated Defense against Bacteria Modulates Infections with Malaria Parasites
Source: PLoS Pathog. 2009 Aug 7;5(8):e1000542. doi: 10.1371/journal.ppat.1000542 (PMC2715215; doi:10.1371/journal.ppat.1000542)

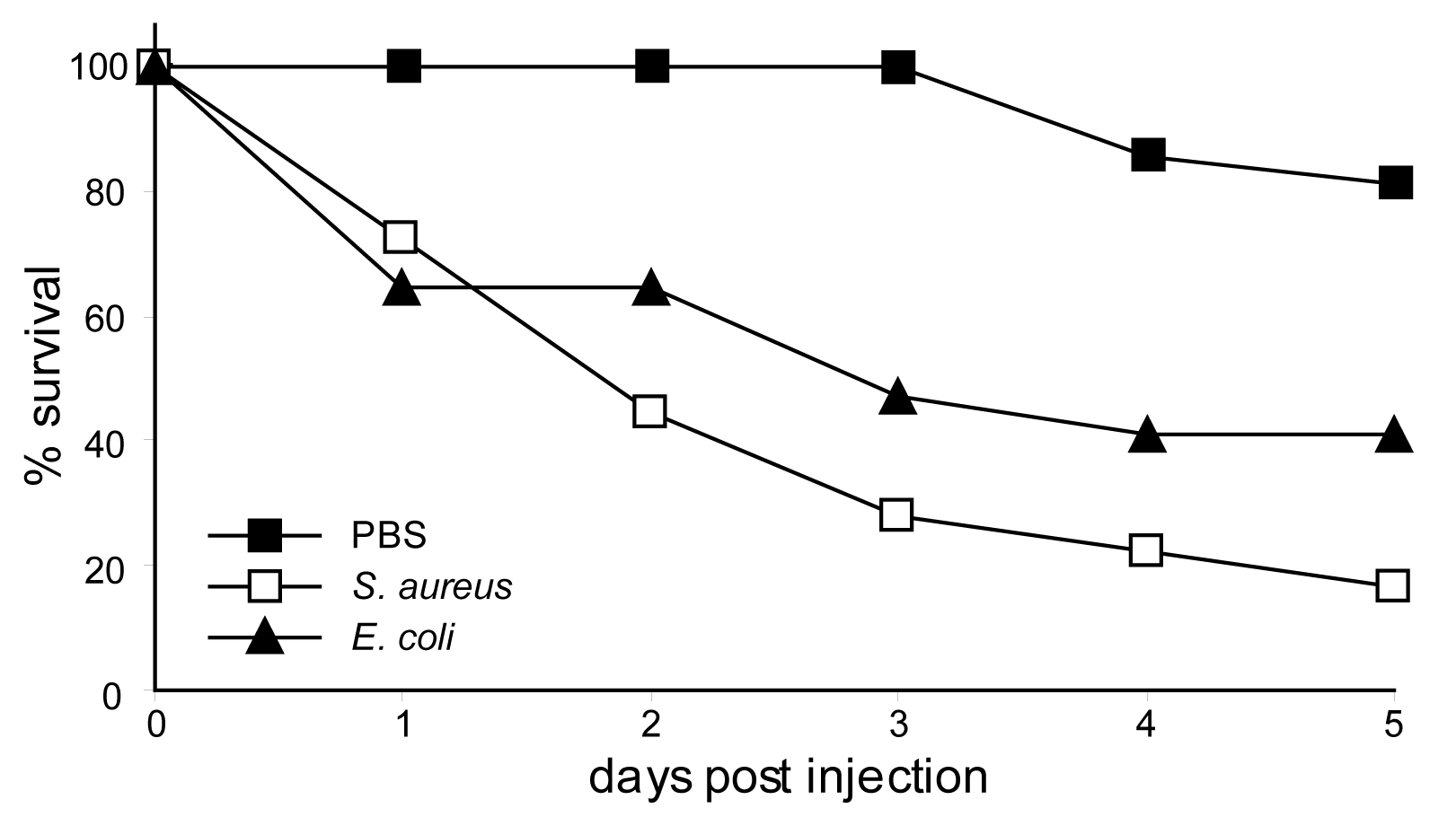

Supplement: Figure S1 — Injection of bacterial suspensions but not with saline alone causes progressive mosquito mortality. Following the indicated injections in female mosquitoes, survival is recorded daily for 5 days. Mortality is minimal and slow after saline injection alone, but rapid and progressive after S. aureus or E. coli injections. (0.23 MB TIF) [file ppat.1000542.s006.tif]

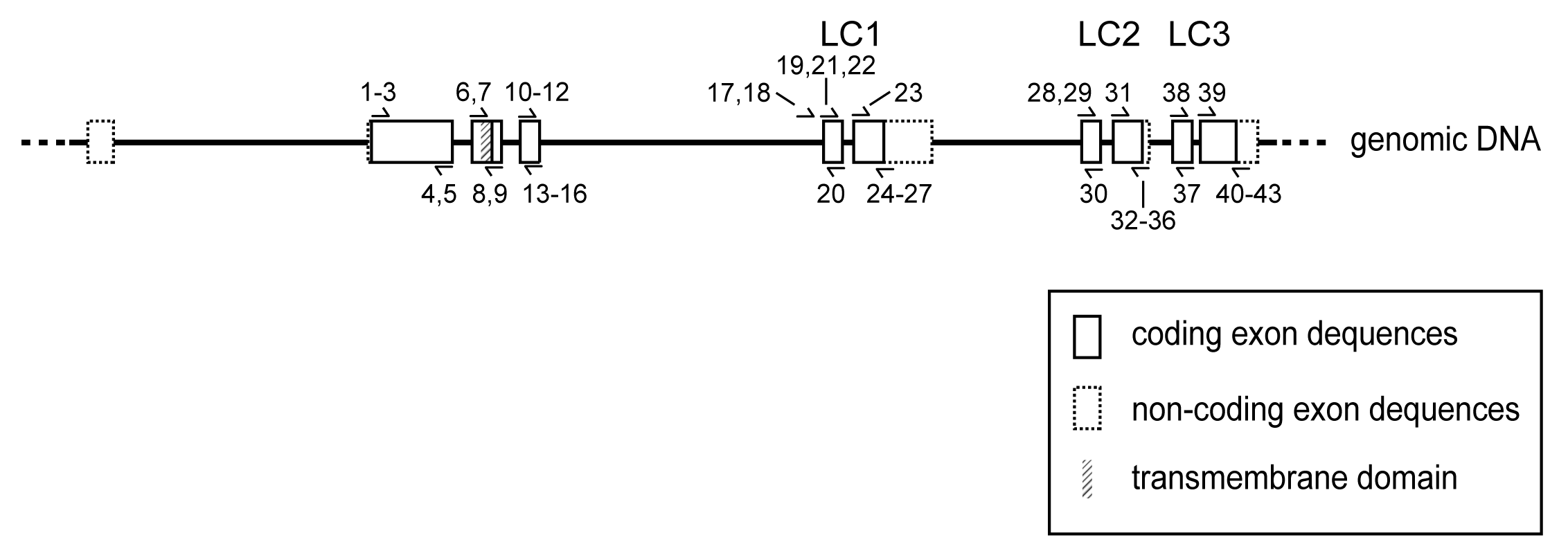

Supplement: Figure S2 — Oligonucleotide primers used in PGRPLC PCR and RT-PCR reactions. The relative position and orientation of primers on the PGRPLC genomic locus is shown. Numbers indicate the primer numbers presented in Table S1. (0.21 MB TIF) [file ppat.1000542.s007.tif]

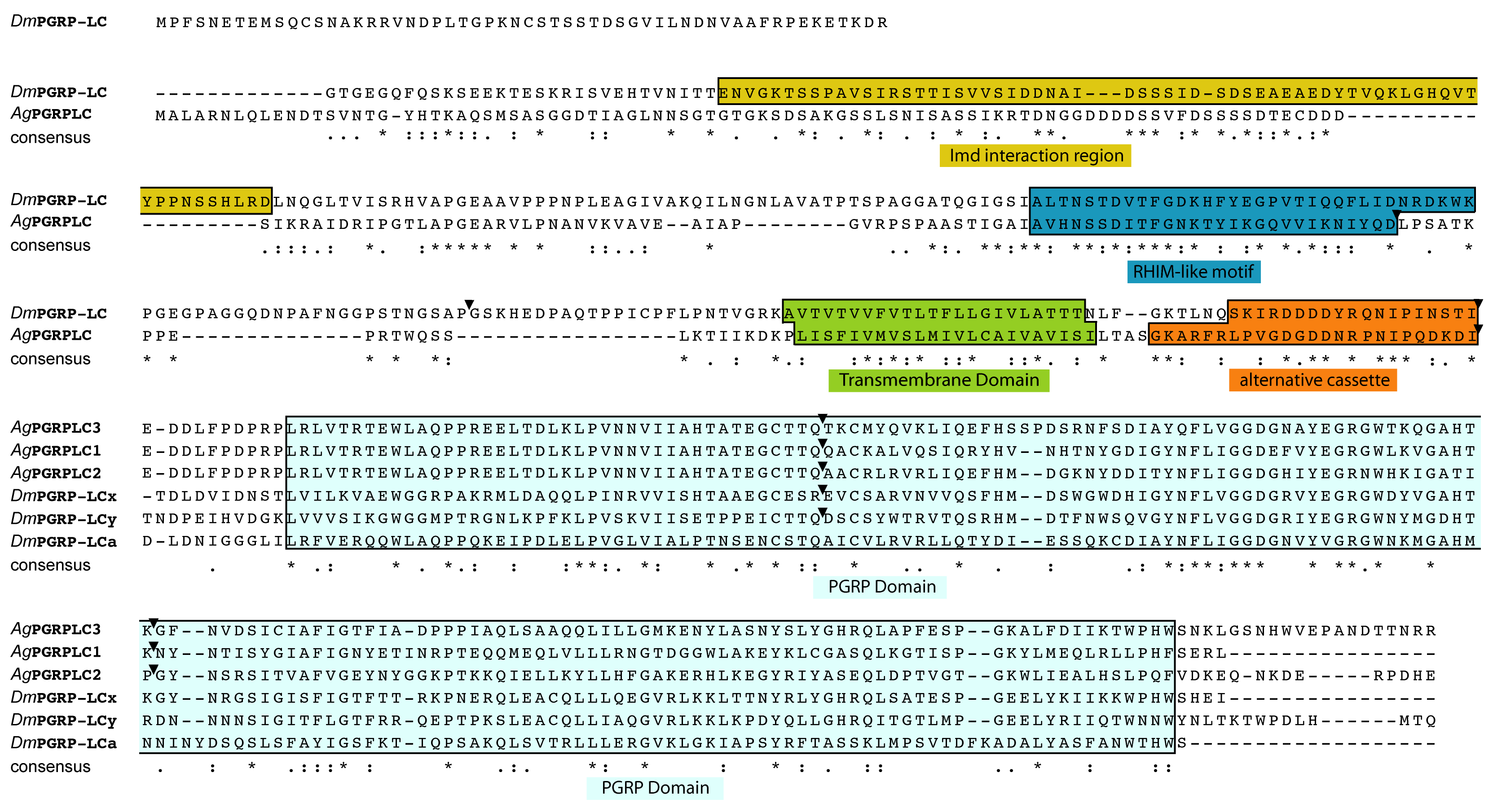

Supplement: Figure S3 — Amino acid alignment of A. gambiae and D. melanogaster PGRPLC main isoforms. The predicted Imd interaction region in Drosophila (yellow), RHIM-like motifs (blue), transmembrane domains, alternative cassettes (orange) and PGRP domains are shown. Intron positions are indicated with black arrowheads. (0.80 MB TIF) [file ppat.1000542.s008.tif]

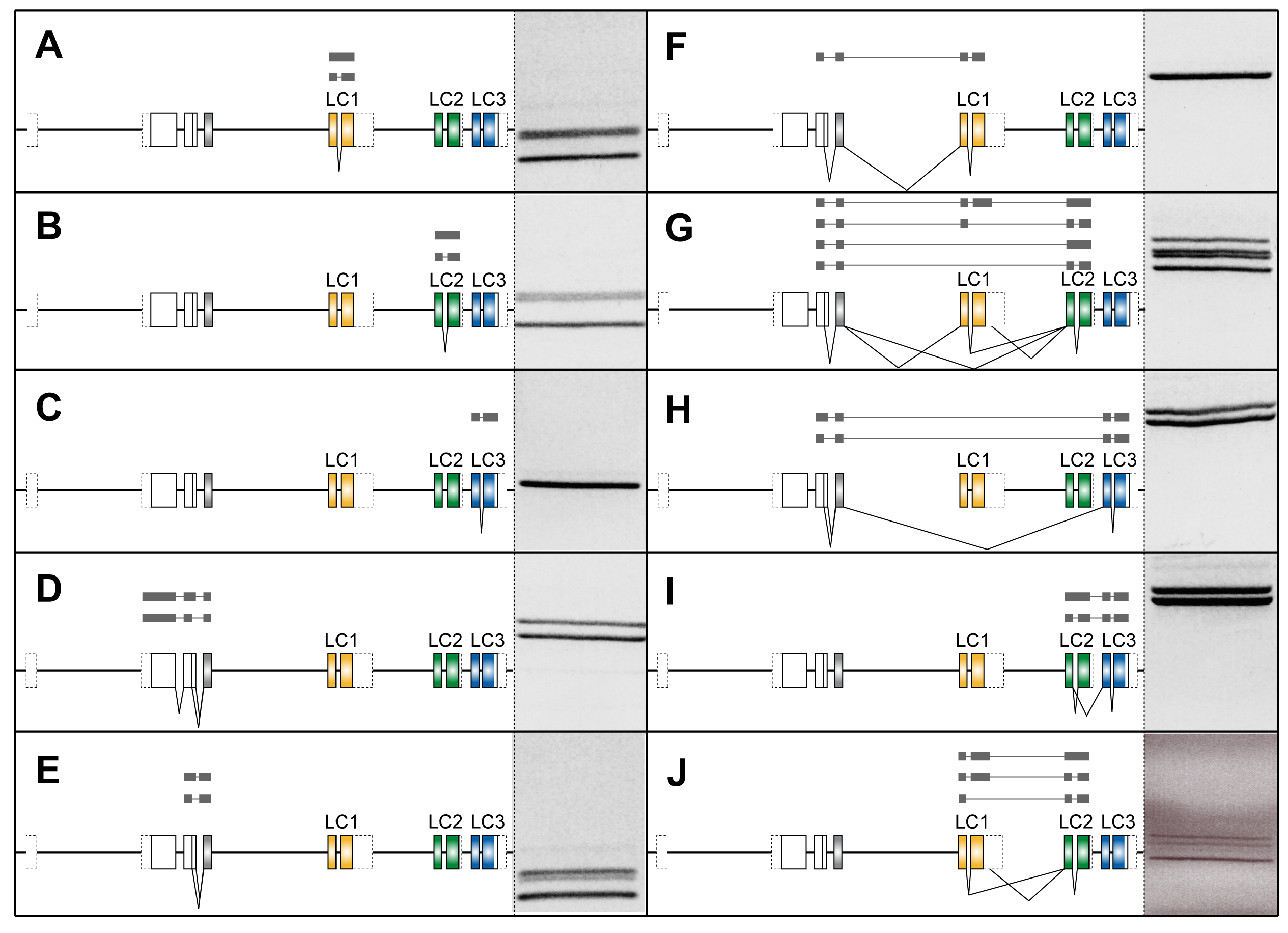

Supplement: Figure S4 — RT-PCR reveals the presence of various PGRPLC isoforms and a pool of unspliced transcripts. Each subpanel (A–J) features a different PCR primer combination and the corresponding gene model prediction. Grey bars above these models represent sequenced RT-PCR products shown on the right side of each panel. (1.78 MB TIF) [file ppat.1000542.s009.tif]

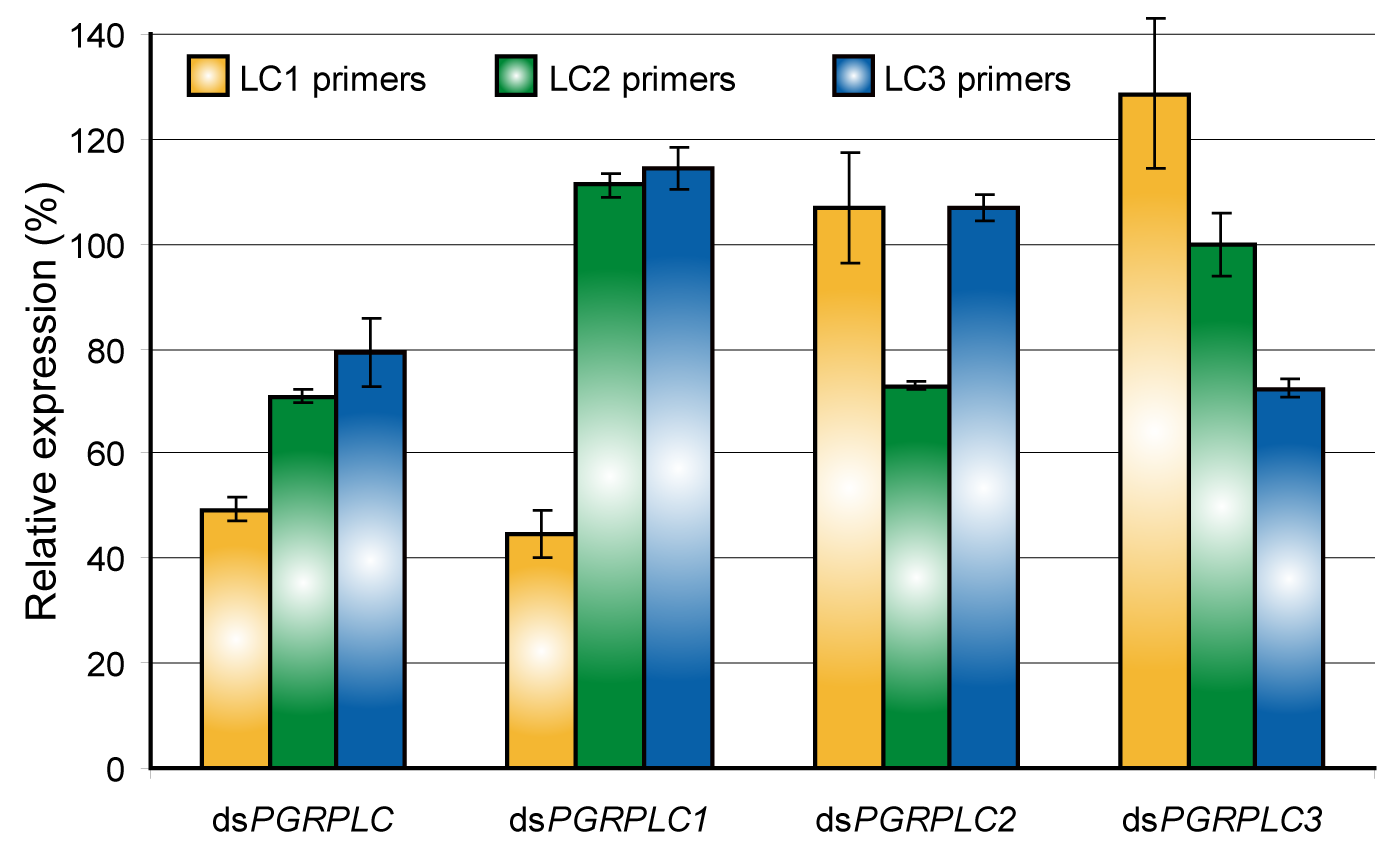

Supplement: Figure S5 — Efficiency of PGRPLC isoform silencing in A. gambiae. Relative percent expression of each of the three main PGRPLC isoforms quantified by qRT-PCR in adult A. gambiae females silenced for the expression of entire PGRPLC gene or each of the three isoforms. A small increase of LC1 transcript levels in LC3 kd mosquitoes may suggest an effect of silencing on the pool of unspliced transcripts, resulting in upregulation of non-targeted transcripts. (0.72 MB TIF) [file ppat.1000542.s010.tif]

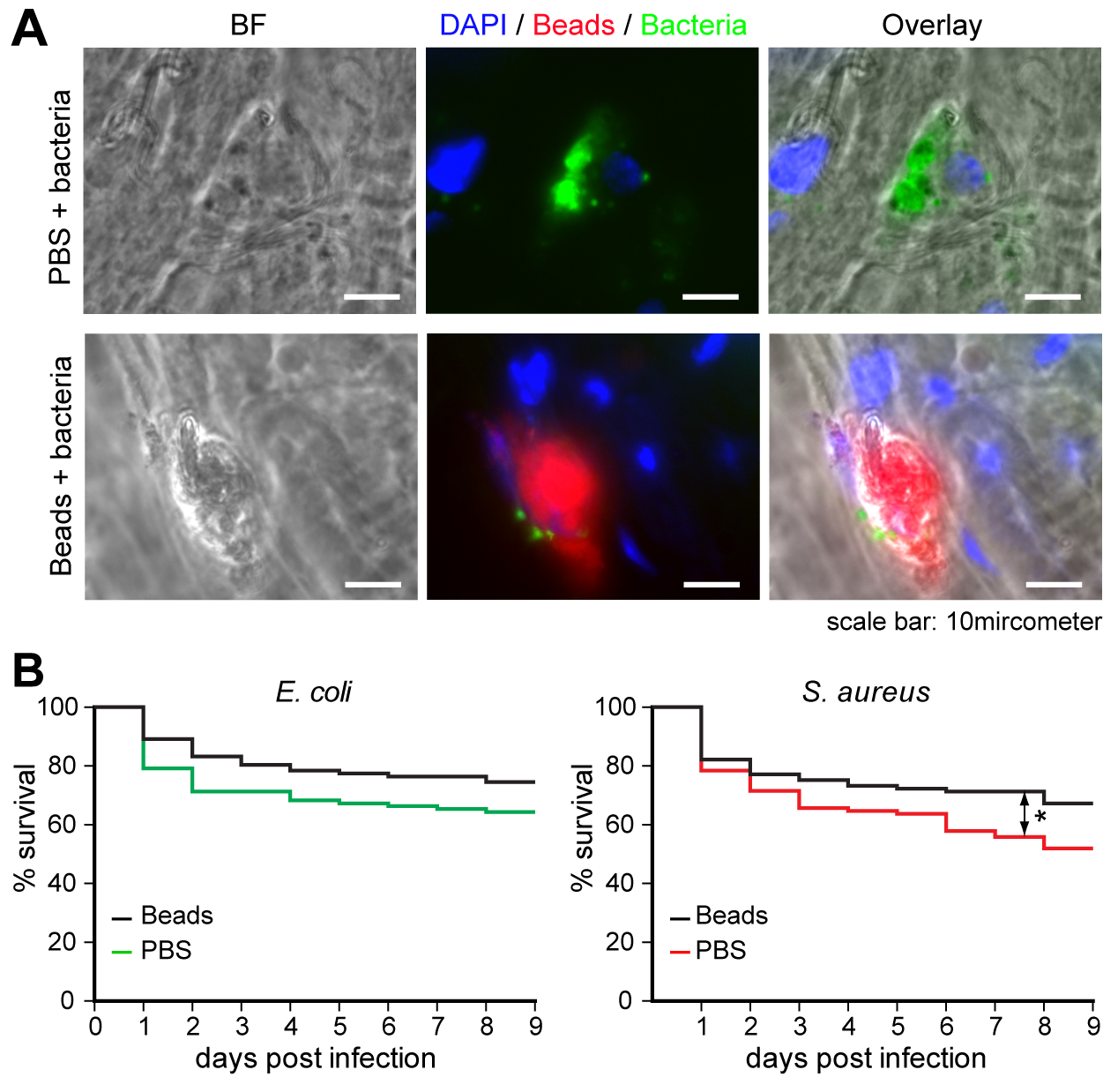

Supplement: Figure S6 — Blocking phagocytosis does not have a major effect on mosquito survival. (A) Indicative microscopic images of hemocytes in A. gambiaeinjected with PBS (top) or Red amine conjugated polystyrene beads (bottom) and re-injected 24 h later with FITCconjugated E. coli. BF, bright field. (B) Percent survival of mosquitoes subjected to the same procedures as in (A) but injected with live instead of fixed E. coli or S. aureus bacteria. Mortality rates of bead-injected mosquitoes were marginally increased compared to control PBS injected and statistically significant (P<0.05) with the Log rank (Mandel-Cox) and the Gehan-Breslow-Wilcoxon tests in S. aureus, but not in E. coli, infections. The presented survival rates are the average of three independent biological replicates. (1.72 MB TIF) [file ppat.1000542.s011.tif]

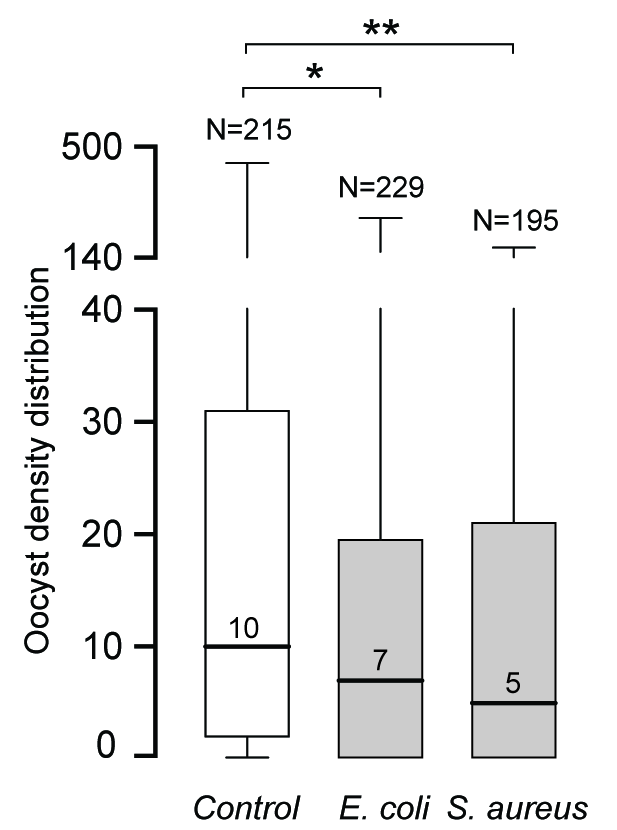

Supplement: Figure S7 — Effect of mosquito midgut infections with different bacteria on P. berghei infection intensities. Median numbers and distribution of P. berghei oocyst intensities in E. coli and S. aureus infected and in control non-infected mosquitoes. Boxes include 50% of the data and whiskers indicate the range in a log10-transformed axis. Median is shown with the bar and number within each box. *, P<0.01; **, P<0.005; N, numbers of mosquitoes. (0.72 MB TIF) [file ppat.1000542.s012.tif]

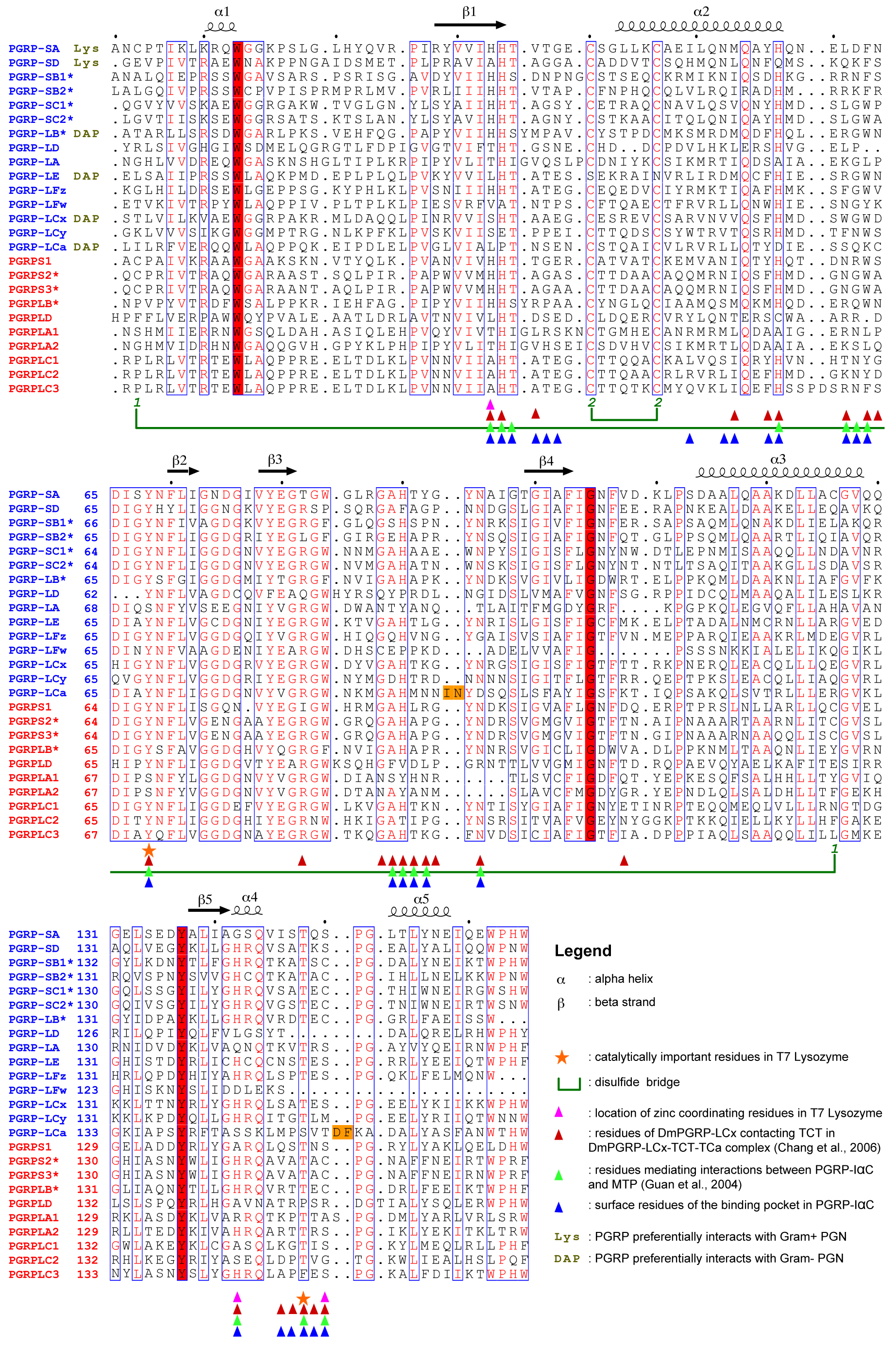

Supplement: Figure S8 — Structure-based multiple alignment of A. gambiae (red) and D. melanogaster (blue) PGRP domains. The secondary structure of PGRP-LCx in the DmPGRP-LCx-TCT-LCa complex is shown above sequences. Asterisks next to PGRP names indicate predicted or experimentally validated amidase activity. Known preferences of PGRPs to Lys and DAP-type PGN are noted. Purple triangles indicate residues implicated in zinc coordination and stars mark catalytically important residues in T7 lysozyme. Brown triangles indicate residues of DmPGRP-LCx that interact with TCT in DmPGRP-LCx-TCT-LCa complex. Green and blue triangles show residues that interact with MTP and shape the PGN binding pocket, respectively, in human PGRP-IaC. Green lines indicate cysteines involved in disulfide bridges. (1.36 MB TIF) [file ppat.1000542.s013.tif]

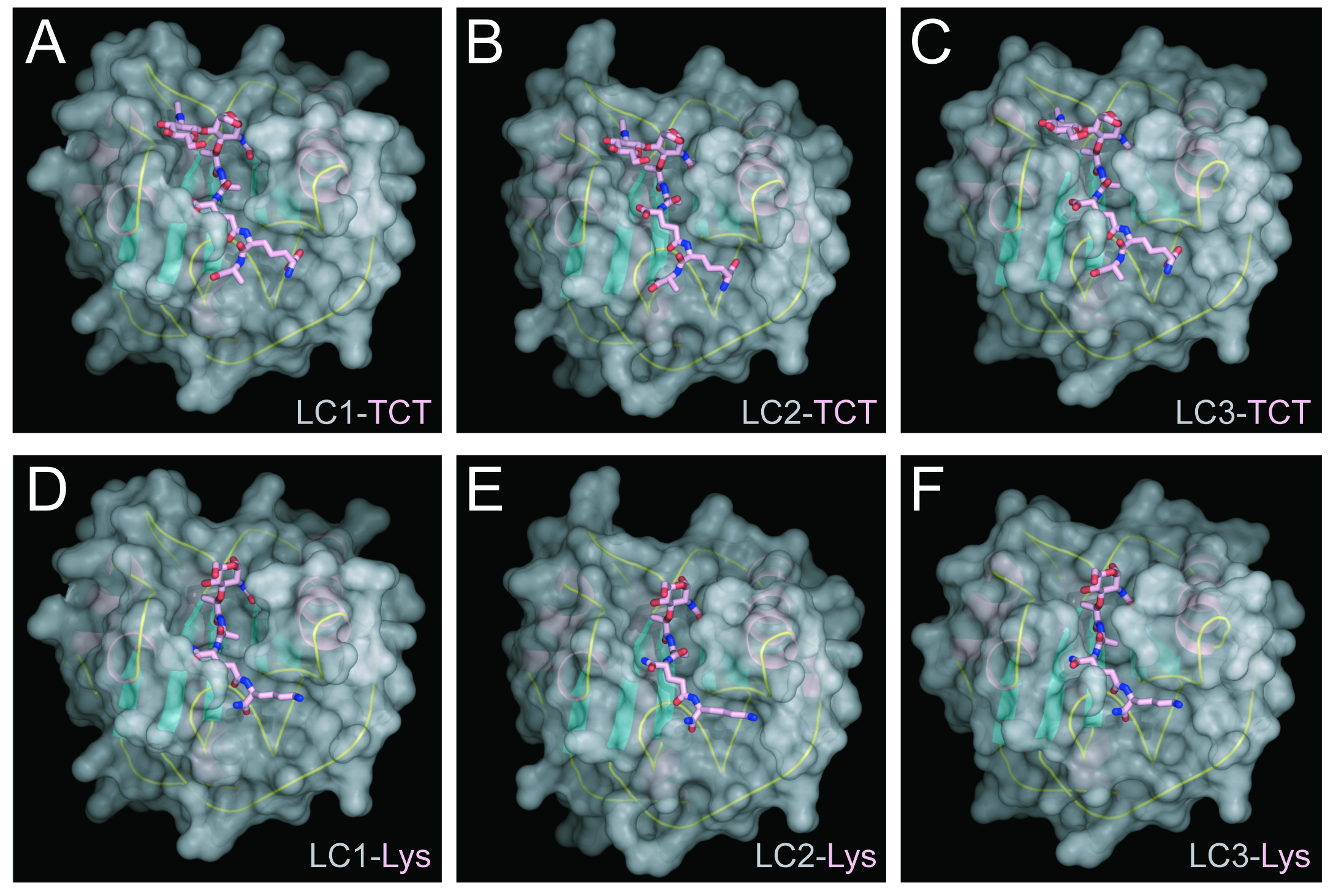

Supplement: Figure S9 — Surface view of AgPGRPLC models in complex with Lys- and DAP-type PGN. The Lys-type muramyl peptide MurNac-L-Ala-D-isoGln-L-Lys (A, B, C) and the DAP-type PGN fragment TCT (D, E, F) are shown in sticks docked to their predicted binding positions in the PGN-binding grooves of AgPGRPLC1, LC2 and LC3, respectively. Secondary-structure elements are visible under semi-transparent surfaces. (6.58 MB TIF) [file ppat.1000542.s014.tif]

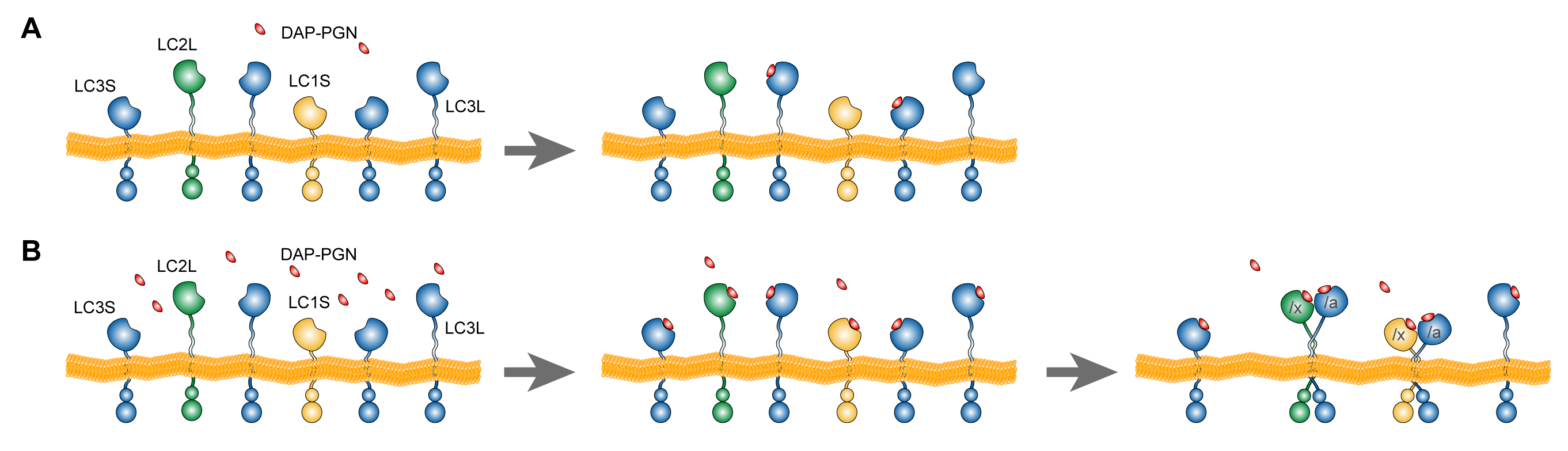

Supplement: Figure S10 — Schematic hypothetical model for the role of PGRPLC3 in modulating immune signaling. (A) Low amounts of PGN are sequestered on the cell surface by the abundant PGRPLC3 that cannot induce dimerization thus dampening the signal. (B) Other PGRPLC isoforms are also engaged in binding PGN that is in high concentrations during high infections and initiate dimerization with PGRPLC3, which thereby locks these isoforms in immunostimulatory dimeric complexes. (0.82 MB TIF) [file ppat.1000542.s015.tif]
